# Recommendations on the structure, personal, and organization of intensive care units

**DOI:** 10.3389/fmed.2023.1196060

**Published:** 2023-06-07

**Authors:** Christian Waydhas, Reimer Riessen, Andreas Markewitz, Florian Hoffmann, Lorenz Frey, Bernd W. Böttiger, Sebastian Brenner, Thorsten Brenner, Teresa Deffner, Matthias M. Deininger, Uwe Janssens, Stefan Kluge, Gernot Marx, Stefan Schwab, Andreas W. Unterberg, Felix Walcher, Thomas van den Hooven

**Affiliations:** ^1^Trauma Intensive Care, Department of Trauma Surgery, University Hospital Essen, Essen, Germany; ^2^Department of Surgery, University Hospital Bergmannsheil, Ruhr-University Bochum, Bochum, Germany; ^3^Medical Intensive Care Unit, Department of Medicine, University of Tübingen, Tübingen, Germany; ^4^Medizinische Geschäftsführung, Deutsche Interdisziplinäre Vereinigung für Intensiv- und Notfallmedizin, Berlin, Germany; ^5^Department of Pediatrics, Dr. Von Hauner Children's Hospital, Ludwig-Maximilians-University, Munich, Germany; ^6^Munich Medical International, Munich, Germany; ^7^Department of Anesthesiology and Intensive Care Medicine, University Hospital of Cologne, Crologne, Germany; ^8^Division of Neonatology and Pediatrics Intensive Care, Department of Pediatrics, University Hospital Carl Gustav Carus, Dresden, Germany; ^9^Department of Anesthesiology and Intensive Care Medicine, University Hospital Essen, University Duisburg-Essen, Essen, Germany; ^10^Department of Anaesthesiology and Intensive Care Medicine, University Hospital Jena, Jena, Germany; ^11^Department of Intensive and Intermediate Care, Medical Faculty, RWTH Aachen University, Aachen, Germany; ^12^Medical Clinic and Medical Intensive Care Medicine, St.-Antonius Hospital, Eschweiler, Germany; ^13^Department of Intensive Care Medicine, University Medical Centre Hamburg-Eppendorf, Heamburg, Germany; ^14^Department of Neurology, University Hospital Erlangen, Friedrich-Alexander-Universität Erlangen-Nürnberg, Eerlangen, Germany; ^15^Department of Neurosurgery, Heidelberg University Hospital, Heidelberg, Germany; ^16^Department of Trauma Surgery, Otto-Von-Guericke University Magdeburg, Magdeburg, Germany; ^17^Pflegedirektion, University Hospital Münster, Münster, Germany

**Keywords:** intensive care medicine, personal, organization, structural requirements, quality of care, recommendation, guideline

## Abstract

**Background:**

Intensive care units (ICU) are central facilities of medical care in hospitals world-wide and pose a significant financial burden on the health care system.

**Objectives:**

To provide guidance and recommendations for the requirements of (infra)structure, personal, and organization of intensive care units.

**Design and setting:**

Development of recommendations based on a systematic literature search and a formal consensus process from a group of multidisciplinary and multiprofessional specialists from the German Interdisciplinary Association of Intensive Care and Emergency Medicine (DIVI). The grading of the recommendation follows the report from an American College of Chest Physicians Task Force.

**Results:**

The recommendations cover the fields of a 3-staged level of intensive care units, a 3-staged level of care with respect to severity of illness, qualitative and quantitative requirements of physicians and nurses as well as staffing with physiotherapists, pharmacists, psychologists, palliative medicine and other specialists, all adapted to the 3 levels of ICUs. Furthermore, proposals concerning the equipment and the construction of ICUs are supplied.

**Conclusion:**

This document provides a detailed framework for organizing and planning the operation and construction/renovation of ICUs.

## Introduction

The first recommendations of the German Interdisciplinary Association of Intensive Care and Emergency Medicine (DIVI) from 2010 (1) and the “Recommendations on basic requirements for intensive care units: structural and organizational aspects” published by the European Society of Intensive Care Medicine (ESICM) in 2011 (2) have exerted their influence over more than 10 years. Since that time the general framework in the public health sector, the economic conditions and medical advancements have significantly changed. Furthermore, various intensive care societies around the world have offered recommendations on their websites. It was the aim of the DIVI to provide a comprehensive and updated guidance and recommendations for the requirements of (infra) structure, personal, and organization of intensive care units. The following manuscript gives an overview over most of the key recommendations. An extensive presentation of the complete text including background information, rationales, references as well as methodology and definitions is provided on the DIVI’s homepage (3).

## Methods

A systematic literature search was performed in PubMed and Epistemonikos in May 2021 (search terms see Supplementary material 1) which identified 2,701 potential publications. Of these, 243 manuscripts were considered relevant und were studied in full text. In addition, relevant publications from their reference lists, recommendations of major intensive care societies and other related documents or webpages were used. The listing of all references used, ordered by recommendation, is presented in Supplementary material 2. The grade of recommendation was determined using the guidelines as suggested by Guyatt et al. (4). A core group (CW, RR, AM, FH, vdH.T), together with the participating sections of the DIVI, suggested a first version of the recommendations (14 video conferences). During a 2-day conference the team of authors completed the recommendations and the whole manuscript was finalized on November 2, 2022.

## Recommendations

In Table 1, all recommendations are summarized and stratified according to the level of the intensive care unit.

**Table 1:** Classification of intensive care units in three levels and minimum requirements for the personal, organizational, and structural features of the respective level.

Level of ICU	Level 1 Basic intensive care	Level 2 Advanced intensive care	Level 3 Comprehensive intensive care
Medical specialty	(Trauma) Surgery Internal medicine Anesthesiology	Surgery Internal medicine Anesthesiology In addition, ≥2 of the following Neurosurgery Orthopedics/Trauma surgery Neurology Internal medicine + cardiology Internal medicine + gastroenterology Gynecology and ≥ 2 of the following Internal medicine + pneumology Vascular surgery Thoracic surgery Urology ENT medicine Ophthalmology Maxillofacial surgery Internal medicine + Hematology/Oncology	Surgery Internal medicine Anesthesiology In addition, ≥ 5 of the following Neurosurgery Orthopedics/Trauma surgery Neurology Internal medicine + cardiology Internal medicine + gastroenterology Gynecology and ≥ 2 of the following Internal medicine + pneumology Vascular surgery Thoracic surgery Urology ENT medicine Ophthalmology Maxillofacial surgery Internal medicine + Hematology/Oncology
Technical equipment	Shock room 24 h CT	Shock room 24 h CT 24 h Endoscopy 24 h PCI 24 h MRI 24 h stroke therapy 24 h bronchoscopy	Shock room 24 h CT 24 h Endoscopy 24 h PCI 24 h MRI 24 h stroke therapy incl. interventional neuroradiology 24 h Bronchoscopy 24 h Interventional radiology
Research and teaching			Structured research in intensive care medicine Structured student teaching
Centre for intensive care (according to the German Federal Joint Committee)			Desirable: Qualification as a center of intensive care (as defined by the German Federal Joint Committee)
Telemedicine	Participation as customer	Participation as customer (optional)	Participation as provider
Number of beds*	≥ 6, all with the option for ventilation	≥ 10 (at the site of the hospital), all with the option of ventilation	≥ 20 (at the site of the hospital), all with the option of ventilation
Qualification medical director	Specialist in intensive care medicine (intensivist)	Specialist in intensive care medicine (intensivist) Working full-time on the intensive care unit and not bound by professional instructions 1 deputy, intensivist	Specialist in intensive care medicine (intensivist) Working full-time on the intensive care unit and not bound by professional instructions 2 deputies, intensivists
Treatment management	Treatment management by an intensivist	Treatment management by an intensivist who performs a large part (>80%) of his or her medical activity in the intensive care unit	Treatment management by an intensivist who performs the majority (>95%) of his or her medical activity in the intensive care unit
Availability and qualification of physicians	An intensivist should carry out at least one daily ward round A physician who has received structured training in intensive care for at least 3 months in this or an intensive care unit of the same or a higher level should be physically present in this intensive care unit 24 h a day, 7 days a week A further physician with education in intensive care medicine (within the framework of training as a specialist in another speciality) of at least 6 months should be present in the hospital and immediately available in the intensive care unit at all times The physician may leave the intensive care unit for a short-term emergency call within the hospital (e.g., reanimation)	An intensivist should carry out at least one daily ward round A physician who has received structured training in intensive care for at least 3 months in this or an intensive care unit of the same or a higher level should be physically present in this intensive care unit 24 h a day, 7 days a week A further physician with education in intensive care medicine (within the framework of training as a specialist in another speciality) of at least 6 months should be present in the hospital and immediately available in the intensive care unit at all times. In addition, such a physician should be physically present in the intensive care unit during core working hours (e.g., at least 7 h between 6 a.m. and 10 p.m.). In addition, an intensivist should be immediately available in the intensive care unit on working days during core working hours. Outside these working hours, an intensivist should be immediately available in the intensive care unit	An intensivist should carry out at least one daily ward round A physician who has received structured training in intensive care for at least 3 months in this or an intensive care unit of the same level should be physically present in this intensive care unit 24 h a day, 7 days a week A further physician with education in intensive care medicine (within the framework of training as a specialist in another speciality) of at least 6 months should be present in the hospital and immediately available in the intensive care unit at all times. In addition, such a physician should be physically present in the intensive care unit during core working hours (e.g., at least 7 h between 6 a.m. and 10 p.m.). In addition, an intensivist should be physically present in the intensive care unit on working days during core working hours (e.g., at least 7 h between 6 a.m. and 10 p.m.) be present at the intensive care unit. Outside these working hours, an intensivist should be immediately available in the intensive care unit
Physician staffing key	According to staffing needs assessment (see details in the recommendation text)		
Nursing staffing key	According to staffing needs assessment (e.g., via INPULS^®^)		
Nursing management	Advanced specialist training in “intensive care and anesthesia” or “Intensive care” A state-recognized training as a ward manager or a degree in nursing management or nursing science or comparable.	Advanced specialist training in “intensive care and anesthesia” or “Intensive care” A state-recognized training as a ward manager, preferably a degree in nursing management or nursing science or comparable	Advanced specialist training in “intensive care and anesthesia” or “Intensive care” A degree in nursing management or nursing science or comparable. But at least a state-recognized training as a ward manager
Nursing qualifications	Rate of specialist training in “intensive care and anesthesia” or “intensive care” of more than >30% of the whole ICU nursing team and within each shift	Rate of specialist training in “intensive care and anesthesia” or “intensive care” of more than >30% of the whole ICU nursing team and within each shift Structured, active and transparent promotion program to increase the share to at least 50%.	Rate of specialist training in “intensive care and anesthesia” or “intensive care” of more than >30% of the whole ICU nursing team and within each shift Structured, active and transparent promotion program to increase the share to at least 50%.
Administrative tasks	Staffing for administrative tasks (ordering, secretarial tasks for the ward, for nurses and physicians)	Staffing for administrative tasks (ordering, secretarial tasks for the ward, for nurses and physicians)	Staffing for administrative tasks (ordering, secretarial tasks for the ward, for nurses and physicians)
Therapist staffing	Physiotherapy (daily)	Physiotherapy (daily) Speech therapy (working days) Occupational therapy (working days, depending on the patients’ requirements)	Physiotherapy (daily) Speech therapy (working days) Occupational therapy (working days)
Hygiene and antibiotic stewardship	Supervision by hygiene officers and hygiene specialists	Supervision by hygiene officers and hygiene specialists Antibiotic stewardship (at least 1 visit per week)	Supervision by hygiene officers and hygiene specialists Antibiotic stewardship (at least 2 visits per week, available on all working days)
Diagnostics in the intensive care unit	X-ray Sonography Transthoracic echocardiography Point-of-care laboratory	X-ray Sonography Transthoracic echocardiography Point-of-care laboratory Bronchoscopy Endoscopy Transesophageal echocardiography	X-ray Sonography Transthoracic echocardiography Point-of-care laboratory Bronchoscopy Endoscopy Transesophageal echocardiography
Availability of therapeutic procedures	Invasive and non-invasive ventilation Highflow oxygen	Invasive and non-invasive ventilation Highflow oxygen Continuous and non-continuous renal replacement procedures	Invasive and non-invasive ventilation Highflow oxygen Continuous and non-continuous renal replacement procedures Plasmapheresis ECMO# ECLS#
IT staff	24/7 availability of specialist IT staff	24/7 availability of IT specialists	24/7 availability of IT specialists
Patient Data Management System (PDMS)	PDMS available	PDMS available	PDMS available
Nutritional therapy	A staff member with a nutritional medicine qualification should be available at least on working days.	A staff member with a nutritional medicine qualification should be available at least on working days. A physician experienced in nutritional medicine, or a nutritionist should be available on a daily basis for consultation in case of special problems.	A staff member with a nutritional medicine qualification should be available at least on working days. A physician experienced in nutritional medicine, or a nutritionist should be available on a daily basis for consultation in case of special problems.
Social service	On working days	On working days	On working days
Spiritual guidance	daily	daily	daily
Ethical case counseling	Ethics committee available (also telemedical) May meet within 48 h on working days and within 72 h on weekends and public holidays.	In-house Ethics Committee May meet within 48 h on working days and within 72 h on weekends and public holidays.	In-house Ethics Committee May meet within 48 h on working days and 72 h on weekends and public holidays.
Palliative care	Palliative care should be available	Palliative care should be available	Palliative care should be available
Cleaning staff	24/7 available	24/7 available	24/7 available
Staff for material and medication supply, cleaning of equipment, equipment maintenance and repair	Available on all working days	Available on all working days	Available on all working days
Ward pharmacist	Permanently assigned pharmacist, telemedical connection also possible, available on all working days At least 1 visit per week	Permanently assigned working hours of a ward pharmacist At least 1 visit per week Availability 24/7	Permanently assigned working hours of a ward pharmacist At least 2 visits per week Availability 24/7
Psychological and psychosocial care (for patients and relatives)	Offer of consultative specialist psychological care for critically ill patients Offer to arrange psychosocial care for relatives	Provision of specialist psychological care for critically ill patients at least on all working days Offer to arrange psychosocial care for relatives	Provision of specialist psychological care for critically ill patients at least on all working days Provision of a psychosocial care service for relatives
Staff welfare and resilience	Timely offer of psychosocial support for medical staff with the help of external psychosocial support and/or Psychosocial support through trained collegial supporters (peers)	Timely offer of psychosocial support for medical staff with to help of external psychosocial support and/or Psychosocial support through trained collegial supporters (peers)	Timely offer of psychosocial support for medical staff with both integrated psychosocial support in the organizational structure and external psychosocial support. Psychosocial support through trained collegial supporters (peers)
Organization	Protocol for the admission, discharge and transfer of patients Regular interprofessional case discussions Internal control of quality indicators relevant for the ICU (at least 2 per year) Participation in external quality benchmarking or an external audit/peer review	Protocol for the admission, discharge and transfer of patients Regular interprofessional case discussions Internal control of at least 4 of the 10 quality indicators per year (completeness of the recorded QI >95%) Participation in external quality benchmarking or an external audit/peer review	Protocol for the admission, discharge and transfer of patients Regular interprofessional case discussions Internal control of at least 6 of the 10 quality indicators per year (completeness of the recorded QI >95%) Participation in external quality benchmarking or an external audit/peer review
# At the site of the hospital Number of beds This recommendation is based on the recommendations of the German Federal Joint Committee on structures in emergency care. For organizational and medical considerations (e.g., intensive care background service) a larger numbers of beds for intensive care medicine appears reasonable. The recommendations from the perspective of intensive care medicine are shown in the last row of this table (see below). This total number of intensive care beds must not be confused with the size of subunits, for which a bed number of 8–12 is recommended			
Number of beds*	≥10, all with the option of ventilation	≥ 20 (at the site), all with the option of ventilation	≥ 50 (at the site), all with the option of ventilation

### Definition of intensive care units

A classification should be made into the following three levels of intensive care units.

- *Basic intensive care* (level 1): In a level 1 intensive care unit, emergency treatments, interventions and therapies can be carried out on a regular basis for which intensive medical monitoring or short-term intensive care is required and sufficient. These intensive care units are able to stabilize patients with disturbed vital signs and treat them temporarily in such a way that the patients can be transferred to an intensive care unit of a higher level within a framework of cooperation agreements or at least written agreements between the partners.
- *Advanced intensive care* (level 2): Level 2 ICUs are equipped to provide complete care for most conservative and surgical ICU patients. Patients for whom necessary medical specialities are not provided (e.g., neurosurgery, cardiac surgery, organ transplant medicine) or who require special types of organ replacement procedures (e.g., ECMO/ECLS) ought to be transferred to level 3 intensive care units within the framework of cooperative agreements.
- *Comprehensive intensive care* (level 3): Level 3 hospitals offer the complete spectrum of intensive care and medical specialities, possibly distributed over various specialized intensive care units. They are adequately staffed and equipped for the care of highly complex patients. Usually, these are university hospitals or large academic teaching hospitals.

In addition, special care providers (e.g., trauma centers, heart centers) must be accounted for and the structural requirements for their intensive care must be defined in analogy.

### Physicians

#### Head of the ICU

An intensive care unit should be led by a physician specialized in intensive care medicine (intensivist)*. In levels 2 and 3, this physician should work full-time in the intensive care unit and hold responsibility for the intensive care medical treatment (grade of recommendation 1A).

* An *intensivist* is a board-certified specialist in anesthesiology, internal medicine, neurology/neurosurgery or surgery with an additional specialized two-year training in intensive care (Germany). In other countries she/he may have acquired another type of specialized board-certified training in intensive care according to the respective national regulations.

#### Qualification and physical presence of physicians

A physician who has received structured training in intensive care for at least 3 months in this or an intensive care unit of the same or a higher level should be physically present in this intensive care unit 24 h a day, 7 days a week (grade of recommendation 1A).

A further physician with education in intensive care medicine (within the framework of training as a specialist in another speciality) of at least 6 months should be present in the hospital and immediately available in the intensive care unit at all times (all levels of ICUs). In addition, in ICUs of levels 2 or 3 such a physician should be physically present in the intensive care unit during core working hours (grade of recommendation 1A).

An intensivist should carry out at least one daily ward round (grade of recommendation 1A).

In ICUs of level 2, in addition, an intensivist should be immediately available in the intensive care unit on working days during core working hours. In level 3 ICUs, an intensivist should be physically present in the intensive care unit on working days during core working hours. Outside these working hours, an intensivist should be immediately available in the intensive care unit (grade of recommendation 1A).

#### Staffing (physicians)

For the position of the head of department and his or her compensation during absence at least 1.3 fulltime equivalents (FTE) are required (grade of recommendation 1A).

In addition, at least 0.7 FTE of physicians per ICU bed is required. The requirements listed under “Qualification and physical presence” must be met (grade of recommendation 1A).

Physicians in their first 3 months of training in the ICU should not be included in the calculation of the physician staffing (grade of recommendation 1C).

In the case of special requirements (e.g., a high number of isolated patients, patients with extensive high-grade burns, polytrauma or extracorporeal organ replacement procedures as well as tasks such as participation in the resuscitation or medical emergency team, shock room coverage, transports of critically ill patients within hospital, complex organizational requirements such as high patient throughput, etc.), a higher number of physicians is required. Therefore, in level 2 and 3 ICUs at least 0.8 FTE physicians (or more, if necessary, according to the local task profile) per ICU bed are required met (grade of recommendation 1C).

#### Physician assistants

Physician assistants facilitate physicians in certain areas of activity (grade of recommendation 2C).

### Nurses

#### Categorization of intensive care patients

Critically ill patients should be classified (on a daily basis) according to the severity of their condition into one of the three Levels of Care (LOC) I, II, or III (grade of recommendation 1C). The allocation of patients to a LOC should be done by means of a validated performance measurement instrument (e.g., INPULS^®^) (grade of recommendation 1C).

#### Management

The ward manager is responsible for the organizational and professional management. The management may refer to an individual person or a team of managers. The ward managers should continue to be, to some part, active in patient care (grade of recommendation 1C).

The ward manager of an intensive care unit should have a specialist training “Intensive care and anesthesia” or “Intensive care.” In addition, she or he should preferably have a degree in nursing management or nursing science or comparable, but at least a state-recognized training in ward management (grade of recommendation 1C).

For the ward manager of an intensive care unit and his or her deputy at least 1.3 FTE for the management tasks alone are required. Additional FTEs are necessary for the active part in patient care. In the case of wards with more than 10 beds, 0.13 FTE should be added for each additional bed (grade of recommendation 1C).

#### Qualification and staffing

The number of nursing staff is to be determined by means of a validated performance measurement instrument (grade of recommendation 1C).

The staffing requirements for the shifts should be calculated on the basis of the LOC levels as averaged over 1 year or a part of a year and be re-evaluated periodically (grade of recommendation 1C).

The calculation should be based on the ratio of nurses to patients and should be 1:3 for patients with LOC I, 1:2 for patients with LOC II and 1:1 for patients with LOC III (grade of recommendation 1A).

The proportion of nurses with additional specialist training in “intensive care and anesthesia” or “intensive care” should be at least 30% of the nursing team in the intensive care unit overall as well as in each shift (grade of recommendation 1C). Measures should be taken to increase their proportion to at least 50% (grade of recommendation 1C).

For the on-the-job training of new nurses and nurses who are in the specialist training for “intensive care and anesthesia” or “intensive care,” an additional 1.3 FTEs per 50 nurses should be provided (grade of recommendation 1C).

The on-the-job training should be carried out in a structured manner by trained nursing staff who are qualified to provide practical guidance (grade of recommendation 1C).

The duration of the on-the-job training for new staff without previous experience in intensive care should be at least 3 months (grade of recommendation 1C).

Nurses, who are on-the-job training should not be included in the calculation for the required patient to nurse ratio (grade of recommendation 1C).

#### Advanced practice nurses

Advanced Practice Nurses (APN) should be employed (grade of recommendation 2C).

#### Release from workload

To release the nursing staff from workload, approved technical and digital aids should be available and used as completely as possible (grade of recommendation 1C).

### Therapists

#### Staffing of physiotherapists, speech therapists, occupational therapists

Physiotherapeutic treatments should be provided for patients in intensive care on a daily basis. The average duration of treatment should be 30 min at the bedside (grade of recommendation 1C).*.

Speech therapy should be ensured on all working days for level 2 and 3 ICUs (grade of recommendation 1C).

Occupational therapy should be ensured on all working days for level 2 and 3 ICUs (grade of recommendation 1C).

* Further information on the duration of physiotherapy sessions and the staffing is given in Supplementary material 3.

### Staffing—other professional groups

#### Hygiene and microbiology

Intensive care units must be supervised by hygiene officers and hygiene specialists in accordance with the current German guidelines of the Robert Koch Institute (grade of recommendation 1A).

#### Antibiotic stewardship

A program for the rational use of antibiotic therapy should be set up in every intensive care unit and its clinical results as well as the use of antibiotics, the occurrence of nosocomial infections and multi-resistant infectious agents should be monitored and documented (grade of recommendation 1A).

#### Ward pharmacist

A ward pharmacist should be a permanent member in direct patient care of the interprofessional treatment team in the intensive care unit (grade of recommendation 1A).

At least once a week (ICU level 1 and 2) or twice a week (ICU level 3) a ward pharmacist should participate in an interprofessional visit in person (hospital levels 1, 2, 3) or by means of telemedicine (ICU level 1) (grade of recommendation 1B).

A pharmacist should be available on call at all times (ICU levels 2 and 3) (grade of recommendation 1C).

The FTE of a ward pharmacist allocated to the intensive care unit should be sufficient to allow for basic professional support activities in addition to visiting activities. These include, among others, interaction and safety checks, participation in the development and implementation of therapeutic guidelines and drug-related applications, assessment of drug consumption and analysis of drug expenditure with the aim of optimizing the use of resources in terms of quality assurance (grade of recommendation 1B).

The manufacturers of medication and the distributors of medication databases should develop and implement, in cooperation with patient data management systems, automated interaction, dosage and safety checks using modern digital technologies, including telematics applications (grade of recommendation 1C).

A training curriculum for pharmacists in intensive care should be developed by the pharmacists societies in cooperation with medical societies (grade of recommendation 1C).

#### Psychological care for patients

Specialist psychological care should be available to all critically ill patients on an as-needed basis, at least on working days (grade of recommendation 1C).

#### Psychosocial care for relatives

For relatives of critically ill patients, an option for psychosocial care should be offered and provided (grade of recommendation 1C).

#### Staff welfare: psychosocial support and strengthening of resilience

Psychosocial support for medical staff should be offered in a timely manner, both as part of the organizational structure and as external psychosocial support (grade of recommendation 1C).

Colleagues (peers) trained in psychosocial support should be available in a timely manner (grade of recommendation 1C).

#### Ethical case consultation

An institutionalized ethics committee should be available and able to meet for ethical case consultation within 48 h on working days, and within 72 h on weekends and public holidays (grade of recommendation 1C).

#### Social service

A social service worker should be available every working day for patients, their relatives and as a contact person for the treatment team (grade of recommendation 1C).

#### Spiritual guidance

Pastoral, confessional and/or spiritual care should be actively offered by the treatment team or made possible at the request of the patient or relatives. This applies in particular in life-threatening situations, and at the expected or foreseeable end of life (grade of recommendation 1C).

#### Palliative care

Physicians working in intensive care should have basic knowledge and skills in palliative medicine. The care of seriously ill and dying patients is the task of every physician (grade of recommendation 1C).

Co-treatment by a palliative care physician should be available. This co-treatment can be provided through integrative, consultative or mixed models (grade of recommendation 1C).

#### Nutritional therapy

A staff member with a nutritional medical qualification should be available at least on all working days (grade of recommendation 2C).

Co-care by nutritionists or dieticians should be available for special problems, especially in the ICUs of levels 2 and 3 (grade of recommendation 2C).

#### Administration

The staffing for administrative tasks (ordering, secretarial tasks for the ward, the nursing staff and the physicians) should be at least 1.3 FTEs. For wards with more than 10 beds, 0.13 FTE should be added for each additional bed (grade of recommendation 1C).

#### Logistics and technology

Additional staff capacity should be planned for the following tasks: Material and medication supply, cleaning of equipment, equipment maintenance, equipment instruction according to regulatory requirements and equipment repair (grade of recommendation 1C).

#### Information technology (IT)

24/7 availability of IT staff should be ensured (grade of recommendation 1C).

#### Cleaning staff

Cleaning staff who are familiar with the specific hygiene requirements of the intensive care unit should clean the intensive care unit on a daily basis. In addition, the qualified cleaning staff should be available 24/7 (grade of recommendation 1C).

### Research and teaching

According to its importance for patient care and the entire health care system, research and teaching in intensive care medicine should be appropriately equipped by all medical schools with specialized academic positions (e.g., professorship) for intensive care medicine as well as with fixed and flexible budgets for research and teaching (grade of recommendation 1C).

To these academic speciality positions research budgets should be assigned. These should include at least a study nurse and a medical or scientific position per 10 beds (grade of recommendation 1C).

An infrastructure should be provided that enables participation in national and international single and multicenter studies and registries (grade of recommendation 1C).

Intensive care medicine at university hospitals should establish intensive care digital networks and databases (grade of recommendation 1C).

Research in intensive care and nursing science should be endowed by the medical faculties with academic positions and budgets for research and teaching in accordance with its importance for the care of critically ill patients and the entire health care system (grade of recommendation 1C).

### Organization and quality assurance

#### Admission criteria

For the admission of patients to the intensive care unit, their discharge to other areas within or outside the hospital and the protocols to be followed in the event of capacity constraints written protocols should be available (grade of recommendation 1C).

#### Quality assurance

Intensive care units should participate in an external quality benchmarking or an external audit/peer review and carry out an internal control of several of the 10 German quality indicators (grade of recommendation 1C).

Additional human resources should be made available for the implementation of quality assurance measures (grade of recommendation 1C).

#### Case discussions

Interprofessional/interdisciplinary case discussions should take place regularly and the results should be documented in a way that is comprehensible for all team members (grade of recommendation 1C).

#### Tasks for the future

The warrant of a sustainable financing of structures for external quality benchmarking (data set definitions, automated data collection, data registers, peer review) should be provided through cooperative activities of the medical societies, the industry, the authorities and other regulatory institutions (grade of recommendation 1C).

Intensive care networks and telematic structures should be implemented (grade of recommendation 2C).

### Technical equipment

The recommendations on the technical equipment can be found on the DIVI homepage (3).

### Construction of ICUs

The recommendations on the construction of ICUs can be found on the DIVI homepage (3).

## Discussion

These recommendations are based on an up-to-date systematic literature search endorsed by a group of experts in intensive care that represent the German Interdisciplinary Association of Intensive Care and Emergency Medicine (DIVI). They cover a wide range of topics such as staffing with physicians, nurses, and therapists as well as with further specialists, e.g., pharmacists, experts in clinical microbiology, psychologists, experts in nutrition, palliative care, and many others. Recommendations, both on quality and quantity, of personal are given and adapted to the different levels of intensive care units in detail.

A potential limitation may be the German perspective. However, intensive care in high-income countries appears to be on a similar level world-wide. Furthermore, the recommendations are not only based on the international literature but also account for existing guidelines and recommendations from many countries with highly developed intensive care medicine. Although the quantity of sound scientific data with respect to organizational aspects of intensive care is constantly rising, there remain many unresolved questions or controversial findings. Therefore, many recommendations, albeit strong, are based much on expert opinion, experience, and common medical knowledge with only little scientific data substantiating them. Future findings may alter some of them.

## Conclusion

This document provides a detailed framework for organizing and planning the operation and construction/renovation of ICUs. A 3-staged level of intensive care units with respect to staffing and equipment, and a 3-staged level of care with respect to the severity of illness are recommended. The recommendations for personal and infrastructure are adapted to the 3 levels of intensive care units.

## Author contributions

All authors contributed to designing, drafting, and reviewing the manuscript.

## Conflict of interest

The authors declare that the research was conducted in the absence of any commercial or financial relationships that could be construed as a potential conflict of interest.

## Publisher’s note

All claims expressed in this article are solely those of the authors and do not necessarily represent those of their affiliated organizations, or those of the publisher, the editors and the reviewers. Any product that may be evaluated in this article, or claim that may be made by its manufacturer, is not guaranteed or endorsed by the publisher.
