# Supplementary material for: Recommendations on the structure, personal, and organization of intensive care units
Source: Front Med (Lausanne). 2023 Jun 7;10:1196060. doi: 10.3389/fmed.2023.1196060 (PMC10325721; doi:10.3389/fmed.2023.1196060)
Supplement: Supplementary file 1 [file Data_Sheet_1.docx]

**Supplementary material 1**

Search terms for the literature search.

The search was performed by Prof. Dr. T. Mathes, previously at the IFOM of the university Witten-Herdecke, now at the university Göttingen.

PubMed

*("intensive care units/organization and ad- ministration"[Majr:NoExp] OR (("intensive care units"[Majr:NoExp] OR "critical care"[Majr:NoExp] OR intensive care unit[tiab] OR intensive care units[tiab] OR ICU[tiab] OR ICUs[tiab] OR (acute[tiab] AND (ward[tiab] OR wards))) AND ("medical staff, hospital/organization and administra- tion"[Majr:NoExp] OR "personnel staffing and scheduling"[Majr:NoExp] OR "work- load"[Majr:NoExp] OR "physicians"[Majr:No- Exp] OR "nurses"[Majr:NoExp] OR "critical care nursing"[Majr:NoExp] OR "physical ther- apists"[Majr:NoExp] OR "occupational thera- pists"[Majr:NoExp] OR "microbiology"[Majr:NoExp] OR "hy- giene"[Majr:NoExp] OR "pharmacology, clini- cal"[Majr:NoExp] OR "psychology"[Majr:NoExp] OR "social work"[Majr:NoExp] OR "pastoral care"[Majr:NoExp] OR "ethics committees, clinical"[Majr:NoExp] OR "palliative care"[Majr:NoExp] OR "hospital design and construction"[Majr:NoExp] OR "equipment and supplies"[Majr:NoExp] OR person- nel[tiab] OR staff[tiab] OR staffing[tiab] OR intensivist*[tiab] OR physician*[tiab] OR nurse*[tiab] OR physiotherapist*[tiab] OR oc- cupational therapist*[tiab] OR microbiolo- gist*[tiab] OR hygienist*[tiab] OR clinical pharmacist*[tiab] OR nutritionist*[tiab] OR psychologist*[tiab] OR ((social[tiab] OR pas- toral[tiab] OR palliative[tiab]) AND (ser- vice[tiab] OR services[tiab] OR care[tiab])) OR ethic*[tiab] OR (cleaning[tiab] AND (staff[tiab] OR personnel[tiab])) OR admin- istration[tiab] OR technology[tiab] OR lo- gistic*[tiab] OR equipment[tiab] OR infrastructure[tiab] OR supply[tiab] OR instru- ment[tiab] OR instruments[tiab] OR instru- mentation[tiab] OR organize[tiab] OR*

*organized[tiab] OR organization[tiab] OR or- ganizing[tiab] OR size[tiab] OR structur*[tiab] OR design[tiab] OR location[tiab] OR plan- ning[tiab] OR quality management[tiab]))) AND („2010/01/01"[EDAT] : „3000"[EDAT]) AND (english[la] OR german[la])*

*NOT (comment [pt] OR editorial [pt] OR letter [pt])
AND (MEDLINE[tiab] OR (systematic[tiab] AND review[tiab]) OR meta-analysis[pt])*

Epistemonikos

(Advanced Search Title/Abstract)

*“intensive care unit” OR “intensive care units” OR ICU OR ICUs OR ward OR wards AND per- sonnel OR staff OR staffing OR intensivist* OR physician* OR nurse* OR physiotherapist* OR “occupational therapist” OR “occupational therapists” OR microbiologist* OR hygienist* OR “clinical pharmacist” OR “clinical pharma- cists” OR nutritionist* OR psychologist* OR “social service” OR “social services” OR “so- cial care” OR “pastoral service” OR “pastoral services” OR “pastoral care” OR “palliative service” OR “palliative services” OR “palliative care” OR ethic* OR “cleaning staff” OR “cleaning personnel” OR administration OR technology OR logistic* OR equipment OR in- frastructure OR supply OR instrument OR in- struments OR instrumentation OR organize OR organized OR organization OR organizing OR size OR structur* OR design OR location OR planning OR “quality management”*
