# Supplementary material for: Recommendations on the structure, personal, and organization of intensive care units
Source: Front Med (Lausanne). 2023 Jun 7;10:1196060. doi: 10.3389/fmed.2023.1196060 (PMC10325721; doi:10.3389/fmed.2023.1196060)
Supplement: Supplementary file 2 [file Data_Sheet_2.docx]

**Supplementary material 2**

**Duration of therapeutic interventions and staff requirements**

The recommended 30 minutes duration of daily physiotherapy are derived from the review article of Sosnoswki et al (Sosnowski, K., et al., Early rehabilitation in the intensive care unit: an integrative literature review. Aust Crit Care, 2015. 28: 216−225) together with the expert opinion. Therefore, a grade of recommendation 1C was given (strong recommendation, with weak evidence/expert opinion). The total of 30 minutes should be diveded in one or two daily sessions. However, the individual requirement may vary (some being longer some being shorter) between patients. The 30 minutes give an average duration of treatment to calculate the number of staff required.  The 30 minutes should be time at the bedside. Time for preparation, documentation, and travel times to and from the patients have to be accounted in addition. This additional time may vary between hospitals depending on the location of the ICUs or the technical equipment used.

In the United Kingdom one therapist for every 4 beds is recommended for level 3 ICUs (Core Standards Working Party of the Joint Professional Standards Committee. Core Standards for Intensive Care Units. 2013.) For a 30-bed ICU this would require 8.5 FTE and an additional 1.5 FTE for assistance personal (Connolly, B.A., et al., Low Levels of Physical Activity During Critical Illness and Weaning: The Evidence-Reality Gap. J Intensive Care Med, 2019. 34: S. 818−827). However, the actual requirements also depend on the complexity of the patient conditions and the case mix.
