# Supplementary material for: Recommendations on the structure, personal, and organization of intensive care units
Source: Front Med (Lausanne). 2023 Jun 7;10:1196060. doi: 10.3389/fmed.2023.1196060 (PMC10325721; doi:10.3389/fmed.2023.1196060)
Supplement: Supplementary file 3 [file Data_Sheet_3.docx]

**Supplementary material 3**

References from the systematic literature search used f or the recommendations. The references are assigned to each chapter of the recommendations.

**Definition of intensive care units**

1. Haupt, M.T., et al., *Guidelines on critical care services and personnel: Recommendations based on a system of categorization of three levels of care*.* Critical Care Medicine, 2003. 31: S. 2677−2683.

2. *College of Intensive Care Medicine of Australia and New Zealand. Minimum standards for intensive care units.* . Zuletzt heruntergeladen [16.11.20212021]; Geladen von: https://www.cicm.org.au/CICM_Media/CICMSite/Files/Professional/IC-1-Minimum-Standards-for-Intensive-Care-Units.pdf.

3. Nederlandse Vereniging voor Anesthesiologie. *Organisatie en werkwijze op intensive care-afdelingen voor volwassenen in Nederland*. 2006. Zuletzt heruntergeladen [16.11.2021]; Geladen von: https://www.nvvc.nl/Kwaliteit/richtlijnen.

4. *Bundesministerium Soziales, Gesundheit, Pflege und Konsumentenschutz. LKF-Modell 2021 für den stationären Bereich* 2021. Zuletzt heruntergeladen [16.11.2021]; Geladen von: https://www.sozialministerium.at/Themen/Gesundheit/Gesundheitssystem/Krankenanstalten/LKF-Modell-2021/Modell-stationaer-2021.html.

5. Gemeinsamer Bundesausschuss. *Regelungen des Gemeinsamen Bundesausschusses zu einem gestuften System von Notfallstrukturen in Krankenhäusern gemäß § 136c Absatz 4 des Fünften Buches Sozialgesetzbuch (SGB V)*. 2020. Zuletzt heruntergeladen [26.4.2022]; Geladen von: https://www.g-ba.de/downloads/62-492-2340/Not-Kra-R_2020-11-20_iK-2020-11-01.pdf.

6. Ministerium für Arbeit Gesundheit und Soziales. *Krankenhausplan Nordrhein-Westfalen 2021*. 2021. Zuletzt heruntergeladen [26.3.2022]; Geladen von: https://www.landtag.nrw.de/portal/WWW/dokumentenarchiv/Dokument/MMV17-5764.pdf.

7. Bundesregierung. *Koalitionsvertrag 2021 – 2025 zwischen der Sozialdemokratischen Partei Deutschlands (SPD), BÜNDNIS 90 / DIE GRÜNEN und den Freien Demokraten (FDP)*. 2021. Zuletzt heruntergeladen [26.3.2022]; Geladen von: https://www.bundesregierung.de/resource/blob/974430/1990812/04221173eef9a6720059cc353d759a2b/2021-12-10-koav2021-data.pdf?download=1.

8. Verband der Universitätsklinika Deutschlands. *Ein Stufenkonzept für die Krankenhausreform*. 2022. Zuletzt heruntergeladen [17.7.2022]; Geladen von: https://www.uniklinika.de/gesundheitspolitischethemen/spitzenversorgung-fuer-alle/.

9. Marx, G., A. Markewitz, and G. van Aalst. *Telemedizin in der Intensivmedizin S1 Leitlinie der DGAI*. 2020. Zuletzt heruntergeladen [17.7.2022]; Geladen von: https://www.awmf.org/uploads/tx_szleitlinien/001-034l_S1_Telemedizin_in-der-Intensivmedizin_2021-01_1.pdf.

10. Gemeinsamer Bundesausschuss. *Beschluss des Gemeinsamen Bundesausschusses über eine Änderung der Zentrums-Regelungen: Zentren in einem intensivmedizinischen digital-gestützten Versorgungsnetzwerk (IDV-Zentren) – Anhang zu den Anlagen 5 und 7*. 2021. Zuletzt heruntergeladen [17.7.2022]; Geladen von: https://www.g-ba.de/downloads/39-261-4720/2021-02-18_Zentrums-Regelungen_IDV-Zentren_BAnz.pdf.

11. Riessen, R., et al., *Diskussionspapier für eine Reform der Krankenhausfinanzierung in Deutschland aus der Perspektive der Intensivmedizin.* Medizinische Klinik - Intensivmedizin und Notfallmedizin, 2020. 115: S. 59−66.

**Physicians**

1. Deutsche Gesellschaft für Anästhesiologie und Intensivmedizin, et al., *Gemeinsame Empfehlungen zur Ausstattung und Organisation interdisziplinärer operativer Intensiveinheiten (IOI)* der Deutschen Gesellschaft für Anästhesiologie und Intensivmedizin und des Berufsverbandes Deutscher Anästhesisten sowie der Deutschen Gesellschaft für Chirurgieund des Berufsverbandes der Deutschen Chirurgen.* Anästh Intensivmed, 2007. 48: S. 230−232.

2. Deutsche Gesellschaft für Anästhesiologie und Intensivmedizin, et al., *Gemeinsame Empfehlung für die Fachgebiete Anästhesiologie und Innere Medizin zur Organisation der Intensivmedizin am Krankenhaus der Deutschen Gesellschaft für Anästhesiologie und Intensivmedizin, der Deutschen Gesellschaft für Innere Medizin, der Deutschen Gesellschaft für Internistische Intensivmedizin, des Berufsverbandes Deutscher Anästhesisten und des Berufsverbandes Deutscher Internisten.* Anästh Intensivmed, 1980. 21: S. 166−167.

3. BfArM. *Operationen- und Prozedurenschlüssel - Version 2021*. 2021. Zuletzt heruntergeladen [13.09.2021]; Geladen von: https://www.dimdi.de/static/de/klassifikationen/ops/kode-suche/opshtml2021/.

4. DIMDI. *OPS 8-98: OPS Regelwerk und Kodierrichtlinien 2022*. 2022. Zuletzt heruntergeladen [19.2.2022]; Geladen von: https://www.icd-code.de/ops/code/8-98.html.

5. Gemeinsamer Bundesausschuss. *Regelungen zur Konkretisierung der besonderen Aufgaben von Zentren und Schwerpunkten gemäß § 136c Absatz 5 SGB V*. 2021. Zuletzt heruntergeladen [13.9.2021]; Geladen von: https://www.g-ba.de/richtlinien/117/.

6. Gemeinsamer Bundesausschuss. *Richtlinie über Maßnahmen zur Qualitätssicherung bei der Durchführung von minimalinvasiven Herzklappeninterventionen gemäß § 136 Absatz 1 Satz 1 Nummer 2 für nach § 108 SGB V zugelassene Krankenhäuser – MHI-RL*. 2021. Zuletzt heruntergeladen [13.9.2021]; Geladen von: https://www.g-ba.de/richtlinien/84/.

7. Intensive Care Society. *Guidelines for the provision of intensive care services (GPICS) second edition (2019).* 2019. Zuletzt heruntergeladen [9.1.2021]; Geladen von: https://www.ficm.ac.uk/sites/ficm/files/documents/2021-10/gpics-v2.pdf.

8. Weiss, M., et al., *Personalbedarfskalkulation “Intensivmedizin“. Überarbeitung der Kalkulationsgrundlagen für den ärztlichen Dienst aus dem Jahr 2012.* Anästh Intensivmed, 2018. 59: S. S458−S481.

9. Neuraz, A., et al., *Patient Mortality Is Associated With Staff Resources and Workload in the ICU: A Multicenter Observational Study.* Crit Care Med, 2015. 43: S. 1587−1594.

10. Rothen, H.U., et al., *Variability in outcome and resource use in intensive care units.* Intensive Care Med, 2007. 33: S. 1329−1336.

11. Gershengorn, H.B., et al., *Association of Intensive Care Unit Patient-to-Intensivist Ratios With Hospital Mortality.* JAMA Intern Med, 2017. 177: S. 388−396.

12. Galloway, M., et al., *The Effect of ICU Out-of-Hours Admission on Mortality: A Systematic Review and Meta-Analysis.* Crit Care Med, 2018. 46: S. 290−299.

13. Huard, P., et al., *Does the full-time presence of an intensivist lead to better outcomes in the cardiac surgical intensive care unit?* J Thorac Cardiovasc Surg, 2020. 159: S. 1363−1375 e7.

14. Kerlin, M.P., et al., *An Official American Thoracic Society Systematic Review: The Effect of Nighttime Intensivist Staffing on Mortality and Length of Stay among Intensive Care Unit Patients.* Am J Respir Crit Care Med, 2017. 195: S. 383−393.

15. Kogan, A., et al., *The Impact of Initiation of an Intensivist-Led Patient Management Protocol on Outcomes After Cardiac Surgery.* J Cardiothorac Vasc Anesth, 2021. 35: S. 2370−2376.

16. Wilcox, M.E., et al., *Do intensivist staffing patterns influence hospital mortality following ICU admission? A systematic review and meta-analyses.* Crit Care Med, 2013. 41: S. 2253−2274.

17. Gershengorn, H.B., et al., *Association of patient-to-intensivist ratio with hospital mortality in Australia and New Zealand.* Intensive Care Med, 2022. 48: S. 179−189.

18. Kerlin, M.P. and P. Caruso, *Towards evidence-based staffing: the promise and pitfalls of patient-to-intensivist ratios.* Intensive Care Med, 2022. 48: S. 225−226.

19. Kommission für Krankenhaushygiene und Infektionsprävention, *Personelle und organisatorische Voraussetzungen zur Prävention nosokomialer Infektionen.* Bundesgesundheitsblatt Gesundheitsforschung Gesundheitsschutz, 2009. 52: S. 951−962.

20. Bundesverband Deutscher Krankenhausapotheker e. V. (ADKA), et al., *Strukturelle und personelle Voraussetzungen für die Sicherung einer rationalen Antiinfektivaverordnung in Krankenhäusern.* Bundesgesundheitsbl, 2020. 63: S. 749–760.

21. Embriaco, N., et al., *High level of burnout in intensivists: prevalence and associated factors.* Am J Respir Crit Care Med, 2007. 175: S. 686−692.

22. Wong, A., et al., *Staff wellbeing in times of COVID-19.* J Intensive Care Soc, 2021. 22: S. 328−334.

23. Rogers, A., W.-T. Hwang, and L. Scott, *The effects of work breaks on staff nurse performance.* JONA (J Nurs Admin), 2004. 34: S. 512–519.

24. Vagts, D., *Organisation u. Management einer Intensivstation*, in *Die Intensivmedizin.*, G. Marx, et al., Editors. 2015, Springer: Berlin, Heidelberg.

25. *Bürgerliches Gesetzbuch*. Zuletzt heruntergeladen [15.9.2021]; Geladen von: https://www.gesetze-im-internet.de/bgb/__630a.html.

26. *Bürgerliches Gesetzbuch.* Zuletzt heruntergeladen [15.9.2021]; Geladen von: https://www.gesetze-im-internet.de/arbzg/.

27. Bundesärztkammer und Kassenärztliche Bundesvereinigung. *Physician Assistant – Ein neuer Beruf im deutschen Gesundheitswesen*. 2017. Zuletzt heruntergeladen [16.9.2021]; Geladen von: https://www.bundesaerztekammer.de/fileadmin/user_upload/downloads/pdf-Ordner/Fachberufe/Physician_Assistant.pdf.

28. Meyer-Treschan, T., et al., *Welchen Beitrag können Physician Assistants zur Gesundheitsversorgung leisten? Eine Abgrenzung zu Ärztinnen und Ärzten in Weiterbildung.* Z Evid Fortbild Qual Gesundhwes, 2021. 164: S. 15−22.

**Nurses**

1. Waydhas, C. and Deutsche Interdisziplinäre Vereinigung für Intensiv- und Notfallmedizin. *Empfehlung zur Struktur und Ausstattung von Intensivtherapiestationen*. 2010. Zuletzt heruntergeladen [15.7.2017]; Geladen von: http://www.divi.de/images/Dokumente/Empfehlungen/Strukturempfehlungen/2011_StrukturempfehlungLangversion.pdf.

2. Waydhas, C., et al., *Intermediate care units : Recommendations on facilities and structure.* Med Klin Intensivmed Notfmed, 2018. 113: S. 33−44.

3. Bundesministerium der Justiz und für Verbraucherschutz and Bundesamts für Justiz. *Verordnung zur Festlegung von Pflegepersonaluntergrenzen in pflegesensitiven Bereichen in Krankenhäusern für das Jahr 2021 (Pflegepersonaluntergrenzen-Verordnung - PpUGV)*. 2020. Zuletzt heruntergeladen [16.1.2022]; Geladen von: https://www.gesetze-im-internet.de/ppugv_2021/PpUGV.pdf.

4. Ministerium für Gesundheit, E., Pflege und Alter des Landes Nordrhein-Westfalen,. *Krankenhausplan NRW*. 2013. Zuletzt heruntergeladen [16.1.2022]; Geladen von: https://broschueren.nordrheinwestfalendirekt.de/broschuerenservice/mgepa/krankenhausplan-nrw-2015/1617

5. Deutsche Gesetzliche Unfallversicherung und Spitzenverband der landwirtschaftlichen Sozialversicherung. *Anforderungen der gesetzlichen Unfallversicherungsträger nach § 34 SGB VII an Krankenhäuser zur Beteiligung am Schwerstverletzungsartenverfahren (SAV)*. 2013. Zuletzt heruntergeladen [16.1.2022]; Geladen von: https://www.dguv.de/medien/landesverbaende/de/med_reha/documents/sav1.pdf.

6. Dreyfus, S. and H. Dreyfus, *A Five-Stage Model of the Mental Activities Involved in Directed Skill Acquisition.* ORC-80-2, Operations Research Center, University of California, Berkeley, 1980.

7. Intensive Care Society. *Levels of Adult Critical Care Second Edition. Consensus Statement*. 2021. Zuletzt heruntergeladen [8.8.2021]; Geladen von: https://www.ics.ac.uk/Society/Policy_and_Communications/Articles/Intensive_Care_2020_and_Beyond.

8. Valentin, A., P. Ferdinande, and E.W.G.o.Q. Improvement, *Recommendations on basic requirements for intensive care units: structural and organizational aspects.* Intensive Care Med, 2011. 37: S. 1575−1587.

9. Zertifizierungskommission für Intensivstationen der Schweizerischen Gesellschaft für Intensivmedizin. *Richtlinien für die Zertifizierung von Intensivstationen*. 2019. Zuletzt heruntergeladen [8.8.2021]. Geladen von: https://www.swiss-icu-cert.ch/files/daten/Dokumente/02_SGI_ZK-IS_Zertifizierung_Richtlinien_2015_Anhang%20I_Kriterien_V16_Revision_2019_DT_190917_FINAL.pdf.

10. Core Standards Working Party of the Joint Professional Standards Committee. *Core Standards for Intensive Care Units*. 2013. Zuletzt heruntergeladen [8.8.2021]; Geladen von: https://www.ficm.ac.uk/sites/default/files/Core%20Standards%20for%20ICUs%20Ed.1%20(2013).pdf.

11. College of intensive Care Medicine of Australia and New Zealand. *Minimum Standards for Intensive Care Units*. 2016. Zuletzt heruntergeladen [9.8.2021]; Geladen von: https://www.cicm.org.au/CICM_Media/CICMSite/Files/Professional/IC-1-Minimum-Standards-for-Intensive-Care-Units.pdf.

12. Glaß, F. and A. Tack, *Personalbedarfsmessung für die Intensivstation.* Pflege Zeitschrift, 2021. 74: S. 21−23.

13. Wilczek, M. and M. Dammer, *Intensivpflege im Spannungsfeld zwischen Personalkosten und Personalbedarf am Beispiel des Intensivpflege- und Leistungserfassungssystems INPULS®*, in *Controlling im Krankenhaus*, W. Zapp, Editor. 2019, Springer Gabler: Wiesbaden. S. 235−298.

14. Miranda, D.R., A. de Rijk, and W. Schaufeli, *Simplified Therapeutic Intervention Scoring System: the TISS-28 items-results from a multicenter study.* Crit Care Med, 1996. 24: S. 64−73.

15. Guenther, U., et al., *[Nursing workload indices TISS-10, TISS-28, and NEMS : Higher workload with agitation and delirium is not reflected].* Med Klin Intensivmed Notfmed, 2016. 111: S. 57−64.

16. Reis Miranda, D., R. Moreno, and G. Iapichino, *Nine equivalents of nursing manpower use score (NEMS).* Intensive Care Med, 1997. 23: S. 760−765.

17. Hoogendoorn, M.E., et al., *Workload scoring systems in the Intensive Care and their ability to quantify the need for nursing time: A systematic literature review.* Int J Nurs Stud, 2020. 101: S. 103−108.

18. Miranda, D.R., et al., *Nursing activities score.* Crit Care Med, 2003. 31: S. 374−382.

19. Margadant, C., et al., *The Nursing Activities Score Per Nurse Ratio Is Associated With In-Hospital Mortality, Whereas the Patients Per Nurse Ratio Is Not.* Crit Care Med, 2020. 48: S. 3−9.

20. *Gesetz zur Weiterentwicklung der Gesundheitsversorgung* Bundesgesetzblatt, 2021. Teil I Nr. 44: S. 2754−2804.

21. *Fachweiterbildung Intensivpflege*. 2022. Zuletzt heruntergeladen [3.1.2022]; Geladen von: https://www.dgni.de/weiterbildung/fachweiterbildung-intensivpflege.html.

22. Deutsche Krankenhausgesellschaft. *DKG-Empfehlung für die Weiterbildung zur Leitung einer Station / eines Bereichs vom 18.6.2019*. 2019. Zuletzt heruntergeladen [18.2.2022]; Geladen von: https://www.dkgev.de/fileadmin/default/Mediapool/2_Themen/2.5._Personal_und_Weiterbildung/2.5.11._Aus-_und_Weiterbildung_von_Pflegeberufen/Leitung_einer_Station_eines_Bereichs/DKG-Empfehlung_2019_06_18.pdf.

23. Driscoll, A., et al., *The effect of nurse-to-patient ratios on nurse-sensitive patient outcomes in acute specialist units: a systematic review and meta-analysis.* Eur J Cardiovasc Nurs, 2018. 17: S. 6−22.

24. Oliveira, A.C., P.C. Garcia, and L.S. Nogueira, *Nursing workload and occurrence of adverse events in intensive care: a systematic review.* Rev Esc Enferm USP, 2016. 50: S. 683−694.

25. Neuraz, A., et al., *Patient Mortality Is Associated With Staff Resources and Workload in the ICU: A Multicenter Observational Study.* Crit Care Med, 2015. 43: S. 1587−1594.

26. Metnitz, B., et al., *Patient volume affects outcome in critically ill patients.* Wien Klin Wochenschr, 2009. 121: S. 34−40.

27. Sakr, Y., et al., *The impact of hospital and ICU organizational factors on outcome in critically ill patients: results from the Extended Prevalence of Infection in Intensive Care study.* Crit Care Med, 2015. 43: S. 519−526.

28. Roos-Blom, M.J., et al., *Association between organizational characteristics and adequate pain management at the intensive care unit.* J Crit Care, 2020. 56: S. 1−5.

29. Verburg, I.W.M., et al., *Is patient length of stay associated with intensive care unit characteristics?* J Crit Care, 2018. 43: S. 114−121.

30. Bagnasco, A., et al., *Unmet nursing care needs on medical and surgical wards: A scoping review of patients' perspectives.* J Clin Nurs, 2020. 29: S. 347−369.

31. Jakimowicz, S., L. Perry, and J. Lewis, *An integrative review of supports, facilitators and barriers to patient-centred nursing in the intensive care unit.* J Clin Nurs, 2017. 26: S. 4153−4171.

32. Bae, S.H., *Intensive care nurse staffing and nurse outcomes: A systematic review.* Nurs Crit Care, 2021. doi: 10.1111/nicc.12588.

33. Rothen, H.U., et al., *Variability in outcome and resource use in intensive care units.* Intensive Care Med, 2007. 33: S. 1329−1336.

34. Intensive Care Society. *Guidelines for the provision of intensive care services (GPICS) second edition (2019).* 2019. Zuletzt heruntergeladen [9.1.2021]; Geladen von: https://www.ficm.ac.uk/standards-research-revalidation/guidelines-provision-intensive-care-services-v2.

35. Iapichino, G., et al., *Proposal of a flexible structural-organizing model for the Intensive Care Units.* Minerva Anestesiol, 2007. 73: S. 501−506.

36. American Thoracic Society*, Fair allocation of intensive care unit resources.* Am J Respir Crit Care Med, 1997. 156: S. 1282−1301.

37. Dawson, S. and J.A. Runk, *Right patient? Right bed? A question of appropriateness.* AACN Clin Issues, 2000. 11: S. 375−385.

38. Pirret, A.M., *Utilizing TISS to differentiate between intensive care and high-dependency patients and to identify nursing skill requirements.* Intensive Crit Care Nurs, 2002. 18: S. 19−26.

39. Wild, C. and M. Narath, *Evaluating and planning ICUs: methods and approaches to differentiate between need and demand.* Health Policy, 2005. 71: S. 289−301.

40. Moreno, R. and D. Reis Miranda, *Nursing staff in intensive care in Europe: the mismatch between planning and practice.* Chest, 1998. 113: S. 752−758.

41. Deutscher Pflegerat e.V. and Aktionsbündnis Patientensicherheit e.V. *Fehlentwicklungen bei der Leiharbeit in der Pflege stoppen*. 2019. Zuletzt heruntergeladen [16.1.2022]; Geladen von: https://deutscher-pflegerat.de/wp-content/uploads/2020/02/dpr-aps_Gemeinsames_Positionspapier_zur_Leiharbeit_in_der_Pflege_1909.pdf.

42. Moyce, S., R. Lash, and M.L. de Leon Siantz, *Migration Experiences of Foreign Educated Nurses: A Systematic Review of the Literature.* J Transcult Nurs, 2016. 27: S. 181−188.

43. Viken, B., E.M. Solum, and A. Lyberg, *Foreign educated nurses' work experiences and patient safety-A systematic review of qualitative studies.* Nurs Open, 2018. 5: S. 455−468.

44. Sozialministerium Baden-Württemberg. *Verordnung des Sozialministeriums über Weiterbildungen für Pflegeberufe in Baden-Württemberg (WVO-Pflegeberufe) vom 22. Oktober 2020*. 2020. Zuletzt heruntergeladen [18.2.2022]; Geladen von: https://www.landesrecht-bw.de/jportal/portal/t/1a1w/page/bsbawueprod.psml/action/portlets.jw.MainAction?p1=7&eventSubmit_doNavigate=searchInSubtreeTOC&showdoccase=1&doc.hl=0&doc.id=jlr-IntensivWeitBiVBW2020pP5&doc.part=S&toc.poskey=#focuspoint.

45. Blanck-Köster, K., et al., *Positionspapier Wissenschaftliche Weiterentwicklung in der Intensivpflege.* Med Klin Intensivmed Notfmed, 2018. 113: S. 672−675.

46. The Faculty of Intensive Care Medicine. *The National Education and Competence Framework for Advanced Critical Care Practitioners*. 2008. Zuletzt heruntergeladen [1.3.2022]; Geladen von:https://ficm.ac.uk/sites/ficm/files/documents/2021‑10/National%20Education%20%26%20Competence%20Framework%20for%20ACCPs.pdf.

47. International Councíl of Nurses. *Guidelines on Advanced Practice Nursing*. 2020. Zuletzt heruntergeladen [3.1.2022]; Geladen von: https://www.icn.ch/system/files/documents/2020-04/ICN_APN%20Report_EN_WEB.pdf.

48. Deutscher Berufsverband für Pflegeberufe. *Advanced Practice Nursing. Pflegerische Expertise für eine leistungsfähige Gesundheitsversorgung*. 2019. Zuletzt heruntergeladen [3.1.2022]; Geladen von: https://www.dbfk.de/media/docs/download/Allgemein/Advanced-Practice-Nursing-Broschuere-2019.pdf.

49. Mendel, S. and J. Feuchtinger, *Aufgabengebiete klinisch tätiger Pflegeexperten in Deutschland und deren Verortung in der internationalen Advanced Nursing Practice.* Pflege Zeitschrift, 2009. 22: S. 208−216.

50. Deutscher Berufsverband für Pflegeberufe, Schweizer Berufsverband der Pflegefachfrauen und Pflegefachmänner, and Österreichischer Gesundheits- und Krankenpflege-Verband. *Advanced Nursing Practice in Deutschland, Österreich und der Schweiz*. 2013. Zuletzt heruntergeladen [3.1.2022]; Geladen von: https://www.dbfk.de/media/docs/download/DBfK-Positionen/ANP-DBfK-OeGKV-SBK_2013.pdf.

51. Özlü, I., *APN Critical Care als interprofessionelle Schnittstelle.* intensiv, 2020. 28: S. 78−84.

52. Kreeftenberg, H.G., et al., *Impact of the Advanced Practice Provider in Adult Critical Care: A Systematic Review and Meta-Analysis.* Crit Care Med, 2019. 47: S. 722−730.

53. Woo, B.F.Y., J.X.Y. Lee, and W.W.S. Tam, *The impact of the advanced practice nursing role on quality of care, clinical outcomes, patient satisfaction, and cost in the emergency and critical care settings: a systematic review.* Hum Resour Health, 2017. 15: S. 63−85.

54. Fry, M., *Literature review of the impact of nurse practitioners in critical care services.* Nurs Crit Care, 2011. 16: S. 58−66.

55. Kleinpell, R.M., et al., *Nurse Practitioners and Physician Assistants in Acute and Critical Care: A Concise Review of the Literature and Data 2008-2018.* Crit Care Med, 2019. 47: S. 1442−1449.

**Therapists**

1. Ringleb, P., M. Köhrmann, and O. Jansen. *Akuttherapie des ischämischen Schlaganfalls, S2e-Leitlinie. in: Deutsche Gesellschaft für Neurologie (Hrsg.) Leitlinien für Diagnostik und Therapie in der Neurologie*. 2021. Zuletzt heruntergeladen [07.02.2022]; Geladen von: www.dgn.org/leitlinien.

2. Schönhofer, B., et al., *[Prolonged Weaning - S2k-Guideline Published by the German Respiratory Society].* Pneumologie, 2019. 73: S. 723−814.

3. DIMDI. *OPS 8-98: OPS Regelwerk und Kodierrichtlinien 2022*. 2022. Zuletzt heruntergeladen [19.2.2022]; Geladen von: https://www.icd-code.de/ops/code/8-98.html.

4. Twose, P., U. Jones, and G. Cornell, *Minimum standards of clinical practice for physiotherapists working in critical care settings in the United Kingdom: A modified Delphi technique.* J Intensive Care Soc, 2019. 20: S. 118−131.

5. Wang, J., et al., *Effects of early mobilization on the prognosis of critically ill patients: A systematic review and meta-analysis.* Int J Nurs Stud, 2020. 110: S. 103−108.

6. Castro-Avila, A.C., et al., *Effect of Early Rehabilitation during Intensive Care Unit Stay on Functional Status: Systematic Review and Meta-Analysis.* PLoS One, 2015. 10: S. e0130722.

7. Welton, C., et al., *Can an interprofessional tracheostomy team improve weaning to decannulation times? A quality improvement evaluation.* Can J Respir Ther, 2016. 52: S. 7−11.

8. Braxenthaler, M., et al., *Manual Physiotherapie in der Intensivmedizin*. 2016, Berlin: Medizinisch Wissenschftliche Verlagsgesellschaft.

9. Haupt, M.T., et al., *Guidelines on critical care services and personnel: Recommendations based on a system of categorization of three levels of care.* Critical care medicine, 2003. 31: S. 2677−2683.

10. Schaller, S., et al., *The Post-ICU Patient - Management of Long-Term Impairments After Critical Illness.* Intensive Care - Emergency Medicine − Anesthesiology, 2020. 2020. 20: S. 275−282.

11. Clarissa, C., et al., *Early mobilisation in mechanically ventilated patients: a systematic integrative review of definitions and activities.* J Intensive Care, 2019. 7: https://doi.org/10.1186/s40560-018-0355-z

12. Doiron, K.A., T.C. Hoffmann, and E.M. Beller, *Early intervention (mobilization or active exercise) for critically ill adults in the intensive care unit.* Cochrane Database Syst Rev, 2018. 3: S. CD010754.

13. Krupp, A., L. Steege, and B. King, *A systematic review evaluating the role of nurses and processes for delivering early mobility interventions in the intensive care unit.* Intensive Crit Care Nurs, 2018. 47: S. 30−38.

14. Lang, J.K., et al., *Clinical Practice Guidelines for Early Mobilization in the ICU: A Systematic Review.* Crit Care Med, 2020. 48: S. e1121−e1128.

15. Anekwe, D.E., et al., *Early rehabilitation reduces the likelihood of developing intensive care unit-acquired weakness: a systematic review and meta-analysis.* Physiotherapy, 2020. 107: S. 1−10.

16. Kayambu, G., R. Boots, and J. Paratz, *Physical therapy for the critically ill in the ICU: a systematic review and meta-analysis.* Crit Care Med, 2013. 41: S. 1543−1554.

17. Tipping, C.J., et al., *The effects of active mobilisation and rehabilitation in ICU on mortality and function: a systematic review.* Intensive Care Med, 2017. 43: S. 171−183.

18. Zang, K., et al., *The effect of early mobilization in critically ill patients: A meta-analysis.* Nurs Crit Care, 2020. 25: S. 360−367.

19. Calvo-Ayala, E., et al., *Interventions to improve the physical function of ICU survivors: a systematic review.* Chest, 2013. 144: S. 1469−1480.

20. Wang, Y.T., et al., *Physical Rehabilitation in the ICU: A Systematic Review and Meta-Analysis.* Crit Care Med, 2022. 50: S. 375−388.

21. Higgins, S.D., et al., *Early mobilization of trauma patients admitted to intensive care units: A systematic review and meta-analyses.* Injury, 2019. 50: S. 1809−1815.

22. Pozuelo-Carrascosa, D.P., et al., *Multimodality respiratory physiotherapy reduces mortality but may not prevent ventilator-associated pneumonia or reduce length of stay in the intensive care unit: a systematic review.* J Physiother, 2018. 64: S. 222−228.

23. Connolly, B., et al., *Physical rehabilitation interventions for adult patients during critical illness: an overview of systematic reviews.* Thorax, 2016. 71: S. 881−90.

24. Ding, N., et al., *What is the optimum time for initiation of early mobilization in mechanically ventilated patients? A network meta-analysis.* PLoS One, 2019. 14: S. e0223151.

25. Potter, K., S. Miller, and S. Newman, *Patient-Level Barriers and Facilitators to Early Mobilization and the Relationship With Physical Disability Post-Intensive Care: Part 2 of an Integrative Review Through the Lens of the World Health Organization International Classification of Functioning, Disability, and Health.* Dimens Crit Care Nurs, 2021. 40: S. 164−173.

26. Sosnowski, K., et al., *Early rehabilitation in the intensive care unit: an integrative literature review.* Aust Crit Care, 2015. 28: S. 216−225.

27. Core Standards Working Party of the Joint Professional Standards Committee. *Core Standards for Intensive Care Units*. 2013. Zuletzt heruntergeladen [8.8.2021]; Geladen von: https://www.ficm.ac.uk/sites/default/files/Core%20Standards%20for%20ICUs%20Ed.1%20(2013).pdf.

28. Connolly, B.A., et al., *Low Levels of Physical Activity During Critical Illness and Weaning: The Evidence-Reality Gap.* J Intensive Care Med, 2019. 34: S. 818−827.

29. OPS-2022 Systematik. *Maschinelle Beatmung und Atemunterstützung über Maske oder Tubus und Beatmungsentwöhnung*. 2022. Zuletzt heruntergeladen [06.02.2022]; Geladen von: https://www.icd-code.de/ops/code/8-718.html.

30. Rheinwald, M., et al., *[Postextubation dysphagia in intensive care patients : Current findings and clinical recommendations].* Anaesthesiologie, 2022. 71: S. 546−555

31. Zuercher, P., C. Moret, and J.C. Schefold, *Dysphagia in the intensive care unit in Switzerland (DICE) - results of a national survey on the current standard of care.* Swiss Med Wkly, 2019. 149: S. w20111.

32. Dylczyk-Sommer, A., *Dysphagia. Part 2: Dysphagia in intensive care patients.* Anaesthesiol Intensive Ther, 2020. 52: S. 233−236.

33. Berney, L., et al., *Acute neurorehabilitation: does a neurosensory and coordinated interdisciplinary programme reduce tracheostomy weaning time and weaning failure?* NeuroRehabilitation, 2014. 34: S. 809−817.

34. Bonvento, B., et al., *Role of the multidisciplinary team in the care of the tracheostomy patient.* J Multidiscip Healthc, 2017. 10: S. 391−398.

35. Royal College of Speech and language therapists. *Position statement: Speech and language therapists working in adult and paediatric critical care units*. 2019. Zuletzt heruntergeladen [17.1.2022]; Geladen von: https://www.rcslt.org/wp-content/uploads/media/docs/clinical-guidance/rcslt-position-statement-critical-care.pdf?la=en&hash=42823C17957D4848818438CBCD5DC3998EF0CDF7.

36. Intensive Care Society. *Guidelines for the provision of intensive care services (GPICS) second edition (2019).* 2019. Zuletzt heruntergeladen [9.1.2022]; Geladen von: https://www.ficm.ac.uk/sites/ficm/files/documents/2021-10/gpics-v2.pdf.

37. Herunter, B. *Logopädie auf Intensivstationen - Personalberechnungsmodell für KAGes-Spitäler*. 2019. Zuletzt heruntergeladen [18.3.2022]; Geladen von: https://online.medunigraz.at/mug_online/pl/ui/$ctx;lang=DE/wbAbs.showThesis?pThesisNr=56797&pOrgNr=1.

38. Rollnik, J.D., et al., *Prolongiertes Weaning in der neurologisch-neurochirurgischen Frührehabilitation.* Der Nervenarzt, 2017. 88: S. 652−674.

39. Barucchieri, L., et al., *DVE general recommendations for occupational therapy in stroke units.* Ergotherapie und Rehabilitation, 2013. 52: S. 27−31.

40. Kieser, B. and S. Thürlemann. *Ergotherapie auf der Intensivstation, Bachelorarbeit 2016*. 2016. Zuletzt heruntergeladen [17.1.2022]. Geladen von: https://core.ac.uk/download/pdf/148756874.pdf

41. Alvarez, E.A., et al., *Occupational therapy for delirium management in elderly patients without mechanical ventilation in an intensive care unit: A pilot randomized clinical trial.* J Crit Care, 2017. 37: S. 85−90.

**Staffing - other professional groups**

# Hygiene & Microbiology

1. Kommission für Krankenhaushygiene und Infektionsprävention, *Empfehlung zum Kapazitätsumfang für die Betreuung von Krankenhäusern und anderen medizinischen Einrichtungen durch Krankenhaushygieniker/innen.* Bundesgesundheitsblatt Gesundheitsforschung Gesundheitsschutz, 2016. 59: S. 1183−1188.

2. Kommission für Krankenhaushygiene und Infektionsprävention, *Personelle und organisatorische Voraussetzungen zur Prävention nosokomialer Infektionen.* Bundesgesundheitsblatt Gesundheitsforschung Gesundheitsschutz, 2009. 52: S. 951−962.

3. Wichmann, D., et al., *Efficacy of introducing a checklist to reduce central venous line associated bloodstream infections in the ICU caring for adult patients.* BMC Infect Dis, 2018. 18: S. 267−275.

# Antibiotic stewardship

1. Bundesverband Deutscher Krankenhausapotheker e. V. (ADKA), et al., *Strukturelle und personelle Voraussetzungen für die Sicherung einer rationalen Antiinfektivaverordnung in Krankenhäusern.* Bundesgesundheitsbl, 2020. 63: S. 749–760.

2. Interagency Coordination Group on Antimicrobial Resistance. *No time to wait: securing the future from drug-resistant infections. Report to the Secretary General of the United Nations*. 2019. Zuletzt heruntergeladen [03.01.2022]; Geladen von: https://www.who.int/publications/i/item/no-time-to-wait-securing-the-future-from-drug-resistant-infections.

3. European Commission. *A European one health action plan against antimicrobial resistance (AMR)*. Zuletzt herunter-geladen [03.01.2022]; Geladen von: https://ec.europa.eu/health/antimicrobial-resistance/eu-action-on-antimicrobial-resistance_de.

4. De With, K., et al. *S3- Leitlinie Strategien zur Sicherung rationaler Antibiotika-Anwendung im Krankenhaus*. 2019. Zuletzt heruntergeladen [03.01.2022]; Geladen von: https://www.awmf.org/leitlinien/detail/ll/092-001.html.

5. Nathwani, D., et al., *Value of hospital antimicrobial stewardship programs [ASPs]: a systematic review.* Antimicrob Resist Infect Control, 2019. 8: S. 35−48.

6. Davey, P., et al., *Interventions to improve antibiotic prescribing practices for hospital inpatients.* Cochrane Database Syst Rev, 2017. 2: S. CD003543.

# Ward pharmacist

1. Hilgarth, H., et al., *Arzneimitteltherapiesicherheit gefördert durch die interprofessionelle Zusammenarbeit von Arzt und Apotheker auf Intensivstationen in Deutschland: Erkenntnisse einer Umfrage.* Med Klin Intensivmed Notfmed, 2022 Published online 8.3.2022, https:// doi.org/10.1007/s00063-022-00898-5)

2. Rothschild, J.M., et al., *The Critical Care Safety Study: The incidence and nature of adverse events and serious medical errors in intensive care.* Crit Care Med, 2005. 33: S. 1694−1700.

3. Latif, A., et al., *National study on the distribution, causes, and consequences of voluntarily reported medication errors between the ICU and non-ICU settings.* Crit Care Med, 2013. 41: S. 389−398.

4. van Mil F, S.M., Verheyen F, Schulz M, *Arzneimittelbezogene Probleme in der öffentlichen Apotheke.* Pharmazeutische Zeitung, 2001. 146: S. 1308−1314.

5. Aly, A., *Definitionen zu Pharmakovigilanz und Arzneimitteltherapiesicherheit.* Arzneiverordnung in der Praxis, 2015. 42: S. 99−104.

6. Wang, T., et al., *Effect of critical care pharmacist's intervention on medication errors: A systematic review and meta-analysis of observational studies.* J Crit Care, 2015. 30: S. 1101−1106.

7. Rivkin, A. and H. Yin, *Evaluation of the role of the critical care pharmacist in identifying and avoiding or minimizing significant drug-drug interactions in medical intensive care patients.* J Crit Care, 2011. 26: S. 104 e1−6.

8. Rice, M., et al., *Pharmacy Personnel's Involvement in Transitions of Care of Intensive Care Unit Patients: A Systematic Review.* J Pharm Pract, 2021. 34: S. 117−126.

9. Lee, H., et al., *Impact on Patient Outcomes of Pharmacist Participation in Multidisciplinary Critical Care Teams: A Systematic Review and Meta-Analysis.* Crit Care Med, 2019. 47: S. 1243−1250.

10. Leape, L.L., et al., *Pharmacist participation on physician rounds and adverse drug events in the intensive care unit.* JAMA, 1999. 282: S. 267−270.

11. Lat, I., et al., *Position Paper on Critical Care Pharmacy Services: 2020 Update.* Crit Care Med, 2020. 48: S. e813−e834.

12. Klopotowska, J.E., et al., *On-ward participation of a hospital pharmacist in a Dutch intensive care unit reduces prescribing errors and related patient harm: an intervention study.* Crit Care, 2010. 14: S. R174.

13. MacLaren, R., et al., *Clinical and economic outcomes of involving pharmacists in the direct care of critically ill patients with infections.* Crit Care Med, 2008. 36: S. 3184−3189.

14. Kessemeier, N., et al., *A new approach on assessing clinical pharmacists' impact on prescribing errors in a surgical intensive care unit.* Int J Clin Pharm, 2019. 41: S. 1184−1192.

15. Murphy, J.E., et al., *American College of Clinical Pharmacy's Vision of the Future: Postgraduate Pharmacy Residency Training as a Prerequisite for Direct Patient Care Practice.* Pharmacotherapy, 2006. 26: S. 722−733.

16. Johnston, K., et al., *Standard of practice in intensive care for pharmacy services'.* Journal of Pharmacy Practice and Research, 2021. 51: S. 165−183.

17. Schumock, G.T., et al., *Economic evaluations of clinical pharmacy services--1988-1995. The Publications Committee of the American College of Clinical Pharmacy.* Pharmacotherapy, 1996. 16: S. 1188−1208.

18. Schumock, G.T., et al., *Evidence of the economic benefit of clinical pharmacy services: 1996-2000.* Pharmacotherapy, 2003. 23: S. 113−132.

19. Preslaski, C.R., et al., *Pharmacist contributions as members of the multidisciplinary ICU team.* Chest, 2013. 144: S. 1687−1695.

20. Leache, L., et al., *Clinical and economic impact of clinical pharmacist interventions regarding antimicrobials on critically ill patients.* Res Social Adm Pharm, 2020. 16: S. 1285−1289.

21. Bosma, B.E., et al., *Pharmacist interventions during patient rounds in two intensive care units: Clinical and financial impact.* Neth J Med, 2018. 76: S. 115−124.

22. Newsome, A.S., T.W. Jones, and S.E. Smith, *Pharmacists Are Associated With Reduced Mortality in Critically Ill Patients: Now What?* Crit Care Med, 2019. 47: S. e1036−e1037.

23. The Faculty of Intensive Care Medicine und The Intensive Care Society. *Guidelines for the Provision of Intensive Care Services*. 2019. Zuletzt heruntergeladen [1.06.2021; Geladen von: https://www.ics.ac.uk/ICS/GuidelinesAndStandards/GPICS_2nd_Edition.aspx.

24. Waydhas, C. and für die Deutsche Interdisziplinäre Vereinigung für Intensiv- und Notfallmedizin. *Empfehlung zur Struktur und Ausstattung von Intensivtherapiestationen*. 2010. Zuletzt heruntergeladen [15.7.2017]; Geladen von: http://www.divi.de/images/Dokumente/Empfehlungen/Strukturempfehlungen/2011_StrukturempfehlungLangversion.pdf.

25. Der Niedersächsische Ministerpräsident. *Nierdersächsisches Krankenhausgesetz*. 2021. Zuletzt heruntergeladen [29.1.2022]; Geladen von: https://www.voris.niedersachsen.de/jportal/portal/t/dt7/page/bsvorisprod.psml/media-type/html?action=controls.jw.MaxMinDocument&showdoccase=1&max=true.

26. Daniels, C., *Chapter 27: Quality assessment of drug therapy*, in *Atkinson's Principles of Clinical Pharmacology. 4th Edition* S. Huang, et al., Editors. 2021, Academic Press. S. 519−535.

27. Strnad, K., et al., *A Systematic Review of ICU and Non-ICU Clinical Pharmacy Services Using Telepharmacy.* Ann Pharmacother, 2018. 52: S. 1250−1258.

28. Meidl, T.M., et al., *Implementation of pharmacy services in a telemedicine intensive care unit.* Am J Health Syst Pharm, 2008. 65: S. 1464−1469.

29. Amkreutz, J., et al., *Medication safety in a German telemedicine centre: Implementation of a telepharmaceutical expert consultation in addition to existing tele-intensive care unit services.* J Telemed Telecare, 2020. 26: S. 105−112.

# Psychological care for patients and relatives

1. Righy, C., et al., *Prevalence of post-traumatic stress disorder symptoms in adult critical care survivors: a systematic review and meta-analysis.* Crit Care, 2019. 23: S. 213−226.

2. Parker, A.M., et al., *Posttraumatic stress disorder in critical illness survivors: a metaanalysis.* Crit Care Med, 2015. 43: S. 1121−1129.

3. Ohtake, P.J., et al., *Physical Impairments Associated With Post-Intensive Care Syndrome: Systematic Review Based on the World Health Organization's International Classification of Functioning, Disability and Health Framework.* Phys Ther, 2018. 98: S. 631−645.

4. Nikayin, S., et al., *Anxiety symptoms in survivors of critical illness: a systematic review and meta-analysis.* Gen Hosp Psychiatry, 2016. 43: S. 23−29.

5. Lee, H., et al., *Impact on Patient Outcomes of Pharmacist Participation in Multidisciplinary Critical Care Teams: A Systematic Review and Meta-Analysis.* Crit Care Med, 2019. 47: S. 1243−1250.

6. Deutsche Gesellschaft für Anästhesie und Intensivmedizin and Deutsche Interdiszíplinäre Vereinigung für Intensivmedizin und Notfallmedizin. *S3-Leitlinie Analgesie, Sedierung und Delirmanagement in der Intensivmedizin*. 2021. Zuletzt heruntergeladen [3.1.2022]; Geladen von: https://www.awmf.org/uploads/tx_szleitlinien/001-012l_S3_Analgesie-Sedierung-Delirmanagement-in-der-Intensivmedizin-DAS_2021-08.pdf.

7. Wade, D.M., et al., *Effect of a Nurse-Led Preventive Psychological Intervention on Symptoms of Posttraumatic Stress Disorder Among Critically Ill Patients: A Randomized Clinical Trial.* JAMA, 2019. 321: S. 665−675.

8. Wade, D.F., et al., *Non-pharmacological interventions to reduce ICU-related psychological distress: a systematic review.* Minerva Anestesiol, 2016. 82: S. 465−478.

9. Villa, G., et al., *Effects of psychological interventions on anxiety and pain in patients undergoing major elective abdominal surgery: a systematic review.* Perioper Med (Lond), 2020. 9: S. 38−44.

10. Sandvik, R.K., et al., *Pain relief from nonpharmacological interventions in the intensive care unit: A scoping review.* J Clin Nurs, 2020. 29: S. 1488−1498.

11. Rosa, R.G., et al., *Effects of post-ICU follow-up on subject outcomes: A systematic review and meta-analysis.* J Crit Care, 2019. 52: S. 115−125.

12. McIlroy, P.A., et al., *The Effect of ICU Diaries on Psychological Outcomes and Quality of Life of Survivors of Critical Illness and Their Relatives: A Systematic Review and Meta-Analysis.* Crit Care Med, 2019. 47: S. 273−279.

13. Gerth, A.M.J., et al., *Changes in health-related quality of life after discharge from an intensive care unit: a systematic review.* Anaesthesia, 2019. 74: S. 100−108.

14. Geense, W.W., et al., *Nonpharmacologic Interventions to Prevent or Mitigate Adverse Long-Term Outcomes Among ICU Survivors: A Systematic Review and Meta-Analysis.* Crit Care Med, 2019. 47: S. 1607−1618.

15. Fuke, R., et al., *Early rehabilitation to prevent postintensive care syndrome in patients with critical illness: a systematic review and meta-analysis.* BMJ Open, 2018. 8: S. e019998.

16. Barreto, B.B., et al., *The impact of intensive care unit diaries on patients' and relatives' outcomes: a systematic review and meta-analysis.* Crit Care, 2019. 23: S. 411−421.

17. Baker, S.C. and J.A. Gledhill, *Systematic Review of Interventions to Reduce Psychiatric Morbidity in Parents and Children After PICU Admissions.* Pediatr Crit Care Med, 2017. 18: S. 343−348.

18. Papathanassoglou, E.D., *Psychological support and outcomes for ICU patients.* Nurs Crit Care, 2010. 15: S. 118−128.

19. Donovan, A.L., et al., *Interprofessional Care and Teamwork in the ICU.* Crit Care Med, 2018. 46: S. 980−990.

20. Deffner, T., et al., *[Psychological care in the intensive care unit : Task areas, responsibilities, requirements, and infrastructure].* Med Klin Intensivmed Notfmed, 2020. 115: S. 205−212.

21. Davidson, J.E., C. Jones, and O.J. Bienvenu, *Family response to critical illness: postintensive care syndrome-family.* Crit Care Med, 2012. 40: S. 618−624.

22. Cameron, J.I., et al., *One-Year Outcomes in Caregivers of Critically Ill Patients.* N Engl J Med, 2016. 374: S. 1831−1841.

23. Davidson, J.E., et al., *Guidelines for Family-Centered Care in the Neonatal, Pediatric, and Adult ICU.* Crit Care Med, 2017. 45: S. 103−128.

24. Chen, C., J. Michaels, and M.A. Meeker, *Family Outcomes and Perceptions of End-of-Life Care in the Intensive Care Unit: A Mixed-Methods Review.* J Palliat Care, 2020. 35: S. 143−153.

25. Zante, B., S.A. Camenisch, and J.C. Schefold, *Interventions in Post-Intensive Care Syndrome-Family: A Systematic Literature Review.* Crit Care Med, 2020. 48: S. e835−e840.

26. Curtis, J.R., et al., *Randomized Trial of Communication Facilitators to Reduce Family Distress and Intensity of End-of-Life Care.* Am J Respir Crit Care Med, 2016. 193: S. 154−162.

# Staff welfare: psychosocial support and strengthening of resilience.

1. Moss, M., et al., *An Official Critical Care Societies Collaborative Statement: Burnout Syndrome in Critical Care Health Care Professionals: A Call for Action.* Am J Crit Care, 2016. 25: S. 368−376.

2. Chuang, C.H., et al., *Burnout in the intensive care unit professionals: A systematic review.* Medicine (Baltimore), 2016. 95: S. e5629.

3. Gualano, M.R., et al., *The Burden of Burnout among Healthcare Professionals of Intensive Care Units and Emergency Departments during the COVID-19 Pandemic: A Systematic Review.* Int J Environ Res Public Health, 2021. 18: S. 8172−8189.

4. Greenberg, N., et al., *Mental health of staff working in intensive care during Covid-19.* Occup Med (Lond), 2021. 71: S. 62−67.

5. Krüsmann, M. and R. Karl, *Zusammenfassung der Ergebnisse: Empfehlungen zur sekundären Prävention*, in *Sekundäre Prävention einsatzbedingter Belastungsreaktionen und -störungen*, W. Butollo, R. Karl, and M. Krüsmann, Editors. 2012, Bundesamt für Bevölkerungsschutz und Katastrophenhilfe. S. 465−483.

6. Richins, M.T., et al., *Early Post-trauma Interventions in Organizations: A Scoping Review.* Front Psychol, 2020. 11: https://doi.org/10.3389/fpsyg.2020.01176

7. Anderson, G.S., et al., *Peer Support and Crisis-Focused Psychological Interventions Designed to Mitigate Post-Traumatic Stress Injuries among Public Safety and Frontline Healthcare Personnel: A Systematic Review.* Int J Environ Res Public Health, 2020. 17. S. 7645; doi:10.3390/ijerph17207645

8. Hinzmann, D., et al., *Peer-Support in der Akutmedizin.* Anästh Intensivmed, 2019. 60: S. 95−101.

9. Hinzmann, D., et al., *„Let‘s talk about … us“. Die Situation an deutschen Klinken mit Blick aus der Anästhesiologie und Intensivmedizin vor der Covid-19-Pandemie. BDA-Befragung zur psychosozialen Unterstützung in der Akutmedizin im Herbst 2019.* Anästh Intensivmed, 2021. 62: S. 92−100.

10. Koll-Krüsmann, M., *Was ist psychosoziale Unterstützung?*, in *Mitarbeitersicherheit ist Patientensicherheit*, R. Strametz, Editor. 2021, Kohlhammer. S. 56−63.

11. Schießl, A., M. Koll-Krüsmann, and A. Hillert, *Zum Umgang mit schwerwiegenden Ereignissen in der ZNA; fehlt einfach nur mehr Resilienz? Prozessorientierte Psychosoziale Unterstützung nach Standards von PSU-Akut e.V.*, C. Lackner, et al., Editors. 2022, Medizinisch Wissenschaftliche Verlagsgesellschaft. S. (in Druck).

12. Frick, E. and A. Schießl, *Resilienz im ärztlichen Berufsalltag fördern.* Zeitschfift für medizinische Ethik, 2015. 61: S. 47−55.

13. Tracy, D.K., et al., *What should be done to support the mental health of healthcare staff treating COVID-19 patients?* Br J Psychiatry, 2020. 217: S. 537−539.

14. Rieckert, A., et al., *How can we build and maintain the resilience of our health care professionals during COVID-19? Recommendations based on a scoping review.* BMJ Open, 2021. 11: S. e043718.

15. Bell, V. and D. Wade, *Mental health of clinical staff working in high-risk epidemic and pandemic health emergencies a rapid review of the evidence and living meta-analysis.* Soc Psychiatry Psychiatr Epidemiol, 2021. 56: S. 1−11.

16. David, E., et al., *COVID-19 Pandemic Support Programs for Healthcare Workers and Implications for Occupational Mental Health: A Narrative Review.* Psychiatr Q, 2022. 93: S. 227−247.

17. Chen, Q., et al., *Mental health care for medical staff in China during the COVID-19 outbreak.* Lancet Psychiatry, 2020. 7: S. e15−e16.

18. Blake, H., et al., *COVID-Well: Evaluation of the Implementation of Supported Wellbeing Centres for Hospital Employees during the COVID-19 Pandemic.* Int J Environ Res Public Health, 2020. 17.

# Ethical case consultation, social service, spiritual guidance, and palliative care

1. White, D.B., et al., *A Randomized Trial of a Family-Support Intervention in Intensive Care Units.* N Engl J Med, 2018. 378: S. 2365−2375.

2. Bundesministerium für Justiz und Verbraucherschutz. *Bürgerliches Gesetzbuch*. 2021. Zuletzt heruntergeladen [29.10.2021; Geladen von: https://www.gesetze-im-internet.de/bgb/.

3. Bennett, F. and S. O'Conner-Von, *Communication Interventions to Improve Goal-Concordant Care of Seriously Ill Patients: An Integrative Review.* J Hosp Palliat Nurs, 2020. 22: S. 40−48.

4. Schneiderman, L.J., *Effect of ethics consultations in the intensive care unit.* Crit Care Med., 2006. 34: S. S359−S363.

5. Meyer-Zehnder, B., et al., *Ethische Fallbesprechungen auf der Intensivstation.* Der Anaesthesist, 2014. 63: S. 477−487.

6. Akademie für Ethik in der Medizin. *Ethikberatung im Gesundheitswesen*. 2021. Zuletzt heruntergeladen [29.10.2021]; Geladen von: https://www.aem-online.de/index.php?id=16.

7. Krones, T., et al., *Advance care planning for the severely ill in the hospital: a randomized trial.* BMJ Support Palliat Care, 2019. 12: S. e411−e423

8. Detering, K.M., et al., *The impact of advance care planning on end of life care in elderly patients: randomised controlled trial.* BMJ, 2010. 340: S. c1345.

9. DeCourcey, D.D., et al., *Advance Care Planning and Parent-Reported End-of-Life Outcomes in Children, Adolescents, and Young Adults With Complex Chronic Conditions.* Crit Care Med, 2019. 47: S. 101−108.

10. Haupt, M.T., et al., *Guidelines on critical care services and personnel: Recommendations based on a system of categorization of three levels of care.* Critical care medicine, 2003. 31: S. 2677−2683.

11. Bundesministerium für Justiz und Verbraucherschutz. *Sozialgesetzbuch (SGB) Fünftes Buch (V) - Gesetzliche Krankenversicherung - (Artikel 1 des Gesetzes v. 20. Dezember 1988, BGBl. I S. 2477) § 39 Krankenhausbehandlung*. 2021. Zuletzt heruntergeladen [29.10.2021]; Geladen von: https://www.gesetze-im-internet.de/sgb_5/__39.html.

12. American College of Critical Care Medicine of the Society of Critical Care Medicine, *Critical care services and personnel: recommendations based on a system of categorization into two levels of care. American College of Critical Care Medicine of the Society of Critical Care Medicine.* Critical care medicine, 1999. 27: S. 422−426.

13. Münch, U., et al. *Empfehlungen zur Unterstützung von belasteten, schwerstkranken, sterbenden und trauernden Menschen in der Corona Pandemie aus palliativmedizinischer Perspektive*. 2020. Zuletzt heruntergeladen [29.10.2021]; Geladen von: https://www.dgpalliativmedizin.de/images/DGP_Unterstuetzung_Belastete_Schwerstkranke_Sterbende_Trauernde.pdf.

14. Nelson, J.E., et al., *Models for structuring a clinical initiative to enhance palliative care in the intensive care unit: a report from the IPAL-ICU Project (Improving Palliative Care in the ICU).* Crit Care Med, 2010. 38: S. 1765−1772.

15. Metaxa, V., et al., *Palliative care interventions in intensive care unit patients.* Intensive Care Med, 2021.

16. Bundesärztekammer, *Grundsätze der Bundesärztekammer zur ärztlichen Sterbebegleitung.* Dtsch.Arztebl., 2011. 108: S. A346−A348.

17. Martin-Loeches, I., et al., *Determinants of time to death in hospital in critically ill patients around the world.* Intensive Care Med, 2016. 42: S. 1454−1460.

18. Fleischmann-Struzek, C., et al., *Hospitalisierung und Intensivtherapie am Lebensende.* Dtsch Arztebl Int, 2019. 116: S. 653−660.

19. Braus, N., et al., *Prospective study of a proactive palliative care rounding intervention in a medical ICU.* Intensive Care Med, 2016. 42: S. 54−62.

20. Gade, G., et al., *Impact of an inpatient palliative care team: a randomized control trial.* J Palliat Med, 2008. 11: S. 180−190.

21. Ma, J., et al., *Early Palliative Care Consultation in the Medical ICU: A Cluster Randomized Crossover Trial.* Crit Care Med, 2019. 47: S. 1707−1715.

22. Zern, E.K., et al., *Utilization of palliative care services for cardiac arrest patients undergoing therapeutic hypothermia: A retrospective analysis.* Resuscitation, 2017. 112: S. 22−27.

23. Zalenski, R.J., et al., *Impact of Palliative Care Screening and Consultation in the ICU: A Multihospital Quality Improvement Project.* J Pain Symptom Manage, 2017. 53: S. 5−12 e3.

24. Okon, T.R., H.S. Vats, and R.A. Dart, *Palliative medicine referral in patients undergoing continuous renal replacement therapy for acute kidney injury.* Ren Fail, 2011. 33: S. 707−717.

25. Adler, K., et al., *Integration der Palliativmedizin in die Intensivmedizin : Systematische Ubersichtsarbeit.* Anaesthesist, 2017. 66: S. 660−666.

26. Aslakson, R., et al., *Evidence-based palliative care in the intensive care unit: a systematic review of interventions.* J Palliat Med, 2014. 17: S. 219−235.

# Nutritional therapy

1. Hoffmann, M., et al., *Risks in Management of Enteral Nutrition in Intensive Care Units: A Literature Review and Narrative Synthesis.* Nutrients, 2020. 13: S. 82−115.

2. Hurt, R.T., et al., *Targeted Physician Education Positively Affects Delivery of Nutrition Therapy and Patient Outcomes: Results of a Prospective Clinical Trial.* JPEN J Parenter Enteral Nutr, 2015. 39: S. 948−952.

3. Hartl, W., et al., *S3-Leitlinie der Deutschen Gesellschaft für Ernährungsmedizin (DGEM) in Zusammenarbeit mit der GESKES und der AKE: Besonderheiten der Überwachung bei künstlicher Ernährung.* Aktuel Ernahrungsmed., 2013. 38: S. e90−e100.

4. Elke, G., et al., *DGEM-Leitlinie: „Klinische Ernährung in der Intensivmedizin“.* Aktuel Ernahrungsmed, 2018. 43: S. 341−408.

5. Cahill, N.E. and D.K. Heyland, *Bridging the guideline-practice gap in critical care nutrition: a review of guideline implementation studies.* JPEN J Parenter Enteral Nutr, 2010. 34: S. 653−659.

6. Turner, P., *Providing optimal nutritional support on the intensive care unit: key challenges and practical solutions.* Proc Nutr Soc, 2010. 69: S. 574−581.

7. Peterson, S.J., et al., *Assessing the influence of registered dietitian order-writing privileges on parenteral nutrition use.* J Am Diet Assoc, 2010. 110: S. 1703−1711.

8. Arney, B.D., et al., *Effect of Registered Dietitian Nutritionist Order-Writing Privileges on Enteral Nutrition Administration in Selected Intensive Care Units.* Nutr Clin Pract, 2019. 34: S. 899−905.

# Administration, logistics, technology, information technology, and cleaning staff

1. American College of Critical Care Medicine of the Society of Critical Care Medicine, *Critical care services and personnel: recommendations based on a system of categorization into two levels of care. American College of Critical Care Medicine of the Society of Critical Care Medicine.* Critical care medicine, 1999. 27: S. 422−426.

2. Haupt, M.T., et al., *Guidelines on critical care services and personnel: Recommendations based on a system of categorization of three levels of care.* Critical care medicine, 2003. 31: S. 2677−2683.

3. Vorstand der Deutschen Gesellschaft für Krankenhaushygiene e. V., *Reinigung in Krankenhäusern – eine Umfrage der DGKH im Jahr 2013.* Hyg Med, 2014. 39: S. 232−235.

4. Eigenstetter, M., et al., *Reinigung im Krankenhaus an der Schnittstelle zwischen Arbeitssicherheit und Hygiene.* Arbeitsmed Sozialmed Umweltmed, 2018. 53: S. 256−263.

5. Deutsche Interdisziplinäre Vereinigung für Intensiv- und Notfallmedizin (DIVI), *Zur Besetzung von Intensiveinheiten mit Pflegepersonal (3.4.1998)* in *Stellungnahmen, Empfehlungen zu Problemen der Intensiv- und Notfallmedizin. 5. Auflage*, A. Karimi and H. Burchardi, Editors. 2005: Köln. S. 125−129.

6. Deutsche Interdisziplinäre Vereinigung für Intensiv- und Notfallmedizin (DIVI), *Zur den Richtzahlen für den Bettenbedarf und die Personalbesetzung von Intensiveinheiten in Akut-Krankenhäusern (20.11.1984)* in *Stellungnahmen, Empfehlungen zu Problemen der Intensiv- und Notfallmedizin. 5. Auflage*, A. Karimi and H. Burchardi, Editors. 2005: Köln. S. 151−157.

7. Deutsche Krankenhausgesellschaft, *Richtlinien für die Organisation der Intensivmedizin in den Krankenhäusern vom 9. September 1974.* Das Krankenhaus, 1974. 66: S. 457−459.

8. Kommission für Krankenhaushygiene und Infektionsprävention beim Robert Koch-Institut (RKI), *Anforderungen an die Hygiene bei der Reinigung und Desinfektion von Flachen.* Bundesgesundheitsblatt Gesundheitsforschung Gesundheitsschutz, 2004. 47: S. 51−61.

**Research and Teaching**

1. John, S., et al., *[Core curriculum Medical intensive care medicine of the German Society of Medical Intensive Care and Emergency Medicine (DGIIN)].* Med Klin Intensivmed Notfmed, 2021. 116: S. 1−45.

2. Sopka, S., et al., *Peer-assisted learning in intensive care undergraduate teaching – concerning value increase for intensive care medicine.* Anästhesiologie Intensivmedizin, 2015. 56: S. 206−215.

3. O'Connor, E. and I. Martin-Loeches, *A blueprint for improving undergraduate education in intensive care medicine.* Crit Care, 2016. 20: S. 212.

4. Reinhart, K. and T. Welte, *Klinische Studien: Abgehängtes Deutschland.* Dt Ärzteblatt, 2022. **119**: S. 706-707.

5. Shokouhbeen, S. 2022, SpringerNature. Persönliche Mitteilung.

6. Ostwald, D., et al. *Fachkräftemangel - Stationärer und ambulanter Bereich bis zum Jahr 2030*. 2010. Zuletzt heruntergeladen [15.8.2022]; Geladen von: https://www.pwc.de/de/gesundheitswesen-und-pharma/assets/fachkraeftemangel.pdf.

7. Salluh, J.I.F., Y.M. Arabi, and A. Binnie, *COVID-19 research in critical care: the good, the bad, and the ugly.* Intensive Care Med, 2021. 47: S. 470−472.

8. Bein, T., et al., *[New challenges for intensive care medicine due to climate change and global warming].* Anaesthesist, 2020. 69: S. 463−469.

9. Dittrich, K., U. Fischer, and B. Hosters, *Systemrelevant und innovativ.* Pflegez, 2021. 74: S. 10−13.

10. Ewers, M., et al., *Forschung in den Gesundheitsfachberufen.* Dtsch Med Wochenschr, 2012. 137: S. S37-S73.

11. Lehmann, Y., et al., *Bestandsaufnahme der Ausbildung in den Gesundheitsfachberufen im europäischen Vergleich (GesinE) – zentrale Ergebnisse und Schlussfolgerungen.* Gesundheitswesen, 2016. 78: S. 407−413.

12. Bundesagentur für Arbeit. *Berichte: Blickpunkt Arbeitsmarkt Mai 2022: Arbeitsmarktsituation im Pflegebereich*. 2022. Zuletzt heruntergeladen [15.8.2022]; Geladen von: https://statistik.arbeitsagentur.de/DE/Statischer-Content/Statistiken/Themen-im-Fokus/Berufe/Generische-Publikationen/Altenpflege.pdf?__blob=publicationFile&v=7.

13. Reuschenbach, B. and I. Darmann-Fink, *Pflege studieren – Intentionen, Strukturen und Erfahrungen*, in *Hochschuldidaktik der Pflege und Gesundheitsfachberufe*, K. Sahmel, Editor. 2018, Springer: Berlin. S. 63-75.

# Organisation, quality assurance, and tasks for the future

1. Nates, J.L., et al., *ICU Admission, Discharge, and Triage Guidelines: A Framework to Enhance Clinical Operations, Development of Institutional Policies, and Further Research.* Crit Care Med, 2016. 44: S. 1553−1602.

2. Nasraway, S.A., et al., *Guidelines on admission and discharge for adult intermediate care units. American College of Critical Care Medicine of the Society of Critical Care Medicine.* Critical care medicine, 1998. 26: S. 607−610.

3. *Guidelines for intensive care unit admission, discharge, and triage. Task Force of the American College of Critical Care Medicine, Society of Critical Care Medicine.* Crit Care Med, 1999. 27: S. 633−638.

4. Haupt, M.T., et al., *Guidelines on critical care services and personnel: Recommendations based on a system of categorization of three levels of care.* Critical care medicine, 2003. 31: S. 2677−2683.

5. Waydhas, C. and für die Deutsche Interdisziplinäre Vereinigung für Intensiv- und Notfallmedizin und deren Sektion Qualität und Ökonomie, *Kerndatensatz Intensivmedizin 2010 der DIVI und DGAI.* DIVI Zeitschrift, 2010. 4: S. 135−137.

6. Braun, J.P., et al., *The German quality indicators in intensive care medicine 2013--second edition.* Ger Med Sci, 2013. 11: S. Doc09.

7. Kumpf, O., et al., *The future development of intensive care quality indicators - a methods paper.* Ger Med Sci, 2020. 18: S. Doc09.

8. Kumpf, O., et al., *Quality indicators in intensive care medicine for Germany - third edition 2017.* Ger Med Sci, 2017. 15: S. Doc10.

9. Kumpf, O., *[Quality indicators in intensive care medicine : Background and practical use].* Med Klin Intensivmed Notfmed, 2021. 116: S. 17−28.

10. Krahwinkel, W., et al., *[10 years of Peer Reviewing: improving treatment by quality indicators from administrative data].* Dtsch Med Wochenschr, 2011. 136: S. 2083−2088.

11. Bundesärztekammer. *Leitfaden Ärztliches Peer Review Verfahren*. 2014. Zuletzt heruntergeladen [23.2.2022]; Geladen von: https://www.bundesaerztekammer.de/fileadmin/user_upload/downloads/Leitfaden_Aerztliches-Peer-Review_2014.pdf.

12. Braun, J., *Intensivmedizinische Peer Reviews. Qualitätsinitiative für Ärzte und Patienten.* Deutsches Ärzteblatt, 2010. 107: S. 1976−1978.

13. Kumpf, O., et al., *Voluntary peer review as innovative tool for quality improvement in the intensive care unit - a retrospective descriptive cohort study in German intensive care units.* Ger Med Sci, 2014. 12: S. Doc17.

14. Ivers, N., et al., *Audit and feedback: effects on professional practice and healthcare outcomes.* Cochrane Database Syst Rev, 2012: S. CD000259.

15. Stollings, J.L., et al., *Best Practices for Conducting Interprofessional Team Rounds to Facilitate Performance of the ICU Liberation (ABCDEF) Bundle.* Crit Care Med, 2020. 48: S. 562−570.

16. Nisbet, G., S. Dunn, and M. Lincoln, *Interprofessional team meetings: Opportunities for informal interprofessional learning.* J Interprof Care, 2015. 29: S. 426−432.

17. Michalsen, A., et al., *Interprofessional Shared Decision-Making in the ICU: A Systematic Review and Recommendations From an Expert Panel.* Crit Care Med, 2019. 47: S. 1258−1266.

18. Braus, N., et al., *Prospective study of a proactive palliative care rounding intervention in a medical ICU.* Intensive Care Med, 2016. 42: S. 54−62.

19. Bennett, F. and S. O'Conner-Von, *Communication Interventions to Improve Goal-Concordant Care of Seriously Ill Patients: An Integrative Review.* J Hosp Palliat Nurs, 2020. 22: S. 40−48.

20. Hillmann, B., et al., *Structure and concept of ICU rounds: the VIS-ITS survey.* Med Klin Intensivmed Notfmed, 2022. 117: S. 276-282.

21. Stow, P.J., et al., *Development and implementation of a high-quality clinical database: the Australian and New Zealand Intensive Care Society Adult Patient Database.* J Crit Care, 2006. 21: S. 133-41.

22. Raffa, J.D., et al., *The Global Open Source Severity of Illness Score (GOSSIS).* Crit Care Med, 2022. 50: S. 1040−1050.

23. Marx, G., A. Markewitz, and G. van Aalst. *Telemedizin in der Intensivmedizin S1 Leitlinie der DGAI*. 2020. Zuletzt heruntergeladen [17.7.2022]; Geladen von: https://www.awmf.org/uploads/tx_szleitlinien/001-034l_S1_Telemedizin_in-der-Intensivmedizin_2021-01_1.pdf.

24. Kalvelage, C., et al., *Decision-Making Authority During Tele-ICU Care Reduces Mortality and Length of Stay-A Systematic Review and Meta-Analysis.* Crit Care Med, 2021. 49: S. 1169−1181.

# Construction of ICUs

1. ARGEBAU Bauministerkonferenz. *Planungshilfe Intensivtherapie. Baulich-funktionelle Anforderungen unter Berücksichtigung psychosozialer Aspekte. Konferenz der für Städtebau, Bau- und Wohnungswesen zuständigen Minister und Senatoren der Länder. Ausschuss für Staatlichen Hochbau – Fachkommission Bau- und Kostenplanung*, Berlin. 2019. Zuletzt heruntergeladen [12.10.2022]; Geladen von: https://www.is-argebau.de/verzeichnis.aspx?id=22338&o=759O5120O5430.

2. Borck, F.K.. *Planung von Einheiten der Intensivmedizin*. In: Kramme R, editor. Medizintechnik: Verfahren - Systeme - Informationsverarbeitung. Berlin, Heidelberg: Springer Berlin Heidelberg; 2017. S. 89−108.

3. Sunder, W., et al., *Bundesinstitut für Bau- Stadt- und Raumforschung (BBSR) im Bundesamt für Bauwesen und Raumordnung. Bauliche Hygiene im Klinikbau. Planungsempfehlungen für die bauliche Infektionsprävention in den Bereichen der Operation, Notfall- und Intensivmedizin*. Forschung für die Praxis, 2018. 13: S. 1−75.

4. Behörde für Gesundheit und Verbraucherschutz der Freien und Hansestadt Hamburg. *Intensiveinheiten in hamburgischen Krankenhäusern: Planungsempfehlung für die bauliche Gestaltung. 2014*. Zuletzt heruntergeladen [12.10.2022]; Geladen von: https://www.hamburg.de/contentblob/4360992/5fd5dc79c26e00cb3e2fa526f421ab50/data/intensiveinheiten-hamburgische-krankenhaeuser.pdf.

5. The Faculty of Intensive Care Medicine und The Intensive Care Society. *Guidelines for the Provision of Intensive Care Services*. 2019. Zuletzt heruntergeladen [1.6.2021]; Geladen von: https://www.ics.ac.uk/ICS/GuidelinesAndStandards/GPICS_2nd_Edition.aspx.

6. Bauministerkonferenz. *Musterbauordnung*. 2019. Zuletzt heruntergeladen [12.10.2022]; Geladen von: https://www.dibt.de/fileadmin/dibt-website/Dokumente/Rechtsgrundlagen/MBO_2019.pdf.

7. Deutsche Gesetzliche Unfallversicherung. *Neu- und Umbauplanung im Krankenhaus unter Gesichtspunkten des Arbeitsschutzes. DGUV Information 207-027*. 2019. Zuletzt heruntergeladen [30.9.2022]; Geladen von: https://publikationen.dguv.de/widgets/pdf/download/article/3461.

8. Bundesministerium der Justiz und für Verbraucherschutz, Bundesamts für Justiz. *Verordnung zur Festlegung von Pflegepersonaluntergrenzen in pflegesensitiven Bereichen in Krankenhäusern für das Jahr 2021 (Pflegepersonaluntergrenzen-Verordnung - PpUGV)*. 2020. Zuletzt heruntergeladen [16.1.2022]; Geladen von: https://www.gesetze-im-internet.de/ppugv_2021/PpUGV.pdf.

9. Valentin, A., and Ferdinande, P., *Recommendations on basic requirements for intensive care units: structural and organizational aspects*. Intensive Care Med. 2011. 37: S. 1575−1587.

10. College of intensive Care Medicine of Australia and New Zealand. *Minimum Standards for Intensive Care Units. College of intensive Care Medicine of Australia and New Zealand*,. 2016. Zuletzt heruntergeladen [9.8.2021]; Geladen von: https://www.cicm.org.au/CICM_Media/CICMSite/Files/Professional/IC-1-Minimum-Standards-for-Intensive-Care-Units.pdf.

11. Intensive Care Society. *Guidelines for the provision of intensive care services (GPICS) second edition (2019).* 2019. Zuletzt heruntergeladen [9.1.2022]; Geladen von: https://www.ficm.ac.uk/sites/ficm/files/documents/2021-10/gpics-v2.pdf.

12. Sektion Krankenhausbau und Raumlufttechnik der Deutschen Gesellschaft für Krankenhaushygiene. *Leitlinie der Deutschen Gesellschaft für Krankenhaushygiene über die Notwendigkeit von Einzelzimmern in Krankenhäusern*. Hygiene & Medizin. 2022. 47: S. 36−41.

13. Stiller, A., et al., *Relationship between hospital ward design and healthcare-associated infection rates: a systematic review and meta-analysis.* Antimicrob Resist Infect Control. 2016. 5: S. 51.

14. Apple, M.. *A Comparative Evaluation of Swedish Intensive Care Patient Rooms*. Health Environments Research & Design Journal. 2014. 7: S. 78−93.

15. Oliver, K. and Kemp. V., *A comparison of nurses' work satisfaction between single-room and multioccupancy adult intensive care units: A mixed-methods integrative review*. Aust Crit Care. 2020. 33: S. 382−389.

16. Kommission für Krankenhaushygiene und Infektionsprävention. *Infektionsprävention im Rahmen der Pflege und Behandlung von Patienten mit 20 übertragbaren Krankheiten: Empfehlung der Kommission für Krankenhaushygiene und Infektionsprävention (KRINKO) beim Robert Koch-Institut*. Bundesgesundheitsblatt, Gesundheitsforschung, Gesundheitsschutz. 2015. 58: S. 1151−1170.

17. Lu, Y., Ossmann, M.M., Leaf, D.E., *Patient visibility and ICU mortality: a conceptual replication*. HERD. 2014. 7: S. 92−103.

18. Kommission fur Krankenhaushygiene und Infektionspravention (KRINKO) beim Robert Koch-Institut. *Anforderungen an die Infektionspravention bei der medizinischen Versorgung von immunsupprimierten Patienten : Empfehlung der Kommission fur Krankenhaushygiene und Infektionspravention (KRINKO) beim Robert Koch-Institut*. Bundesgesundheitsblatt Gesundheitsforschung Gesundheitsschutz. 2021. 64: S. 232−264.

19. Ausschuss für Arbeitsstätten beim Bundesministerium für Arbeit und Soziales. *Technische Regeln für Arbeitsstätten: Raumtemperatur ASR A3.5.* 2022. Zuletzt heruntergeladen [12.10.2022]; Geladen von: https://www.baua.de/DE/Angebote/Rechtstexte-und-Technische-Regeln/Regelwerk/ASR/ASR-A3-5.html.

20. Lenzer, B., et al., *Health effects of heating, ventilation and air conditioning on hospital patients: a scoping review.* BMC Public Health. 2020. 20: S. 1287.

21. Kommission, für Krankenhaushygiene und Infektionsprävention (KRINKO) beim Robert Koch-Institut. *Anforderungen der Hygiene an abwasserfuhrende Systeme in medizinischen Einrichtungen*. Bundesgesundheitsblatt Gesundheitsforschung Gesundheitsschutz. 2020. 63: S. 484−501.

22. Planungsbüro der Berufsgenossenschaft für Gesundheitsdienst und Wohlfahrtspflege und Unfallkasse Nordrhein-Westfalen. Sicheres Krankenhaus. 2016. Zuletzt heruntergeladen [13.10.2022]; Geladen von: https://sikh.rms2cdn.de/files/gesamt-pdf/planungsbuero.pdf.

23. Verbund für Angewandte Hygiene. *VAH-Liste*. 2022. Zuletzt heruntergeladen [13.10.2022]; Geladen von: https://vah-online.de/de/vah-liste.

24. Kramme, R., Uhlig, H., Feigl, H., *Technische Sicherheit von medizintechnischen elektrischen Geräten und Systemen in medizinisch genutzten Räumen*. In: Kramme R, editor. Medizintechnik. Berlin Heidelberg: Springer; 2015. S. 1−11.

25. Flügel, T., *Leitfähiger Fußboden in einem Krankenhaus.* Elektropraktiker. 2007. 61: S. 483−484.

26. Bayerisches Landesamt für Gesundheit und Lebensmittelsicherheit. Spezialeinheit Infektionshygiene. *Erläuterungen zur Checkliste „Versorgung von immunsupprimierten Patienten“.* 2016. *The future of intensive care: delirium should no longer be an issue* https://www.lgl.bayern.de/downloads/gesundheit/hygiene/doc/begleittext_immunsupprimierte_patienten.pdf.

27. Reinke, L., et al., *The importance of the intensive care unit environment in sleep-A study with healthy participants.* J Sleep Res. 2020. 29: S. e12959.

28. Prajapat, B., et al., *Evaluation of Sleep Architecture using 24-hour Polysomnography in Patients Recovering from Critical Illness in an Intensive Care Unit and High Dependency Unit: a Longitudinal, Prospective, and Observational Study*. J Crit Care Med (Targu Mures). 2021- 7: S. 257−266.

29. Luetz, A., et al*., Innovative ICU Solutions to Prevent and Reduce Delirium and Post-Intensive Care Unit Syndrome.* Semin Respir Crit Care Med. 2019. 40: S. 673−686.

30. Darbyshire, J.L., et al., *Mapping sources of noise in an intensive care unit*. Anaesthesia. 2019. 74: S. 1018−1025.

31. Saha, S., et al., *Mapping the impact of ICU design on patients, families and the ICU team: A scoping review.* J Crit Care. 2022. 67: S. 3−13.

32. Kotfis, K., et al., *The future of intensive care: delirium should no longer be an issue.* Crit Care. 2022. 26: S. 200.

33. Kommission fur Krankenhaushygiene und Infektionspravention (KRINKO) beim Robert Koch-Institut. *Pravention postoperativer Wundinfektionen.* Bundesgesundheitsblatt Gesundheitsforschung Gesundheitsschutz. 2018. 61: S. 448−473.

34. Sektion Krankenhausbau und Raumlufttechnik der Deutschen Gesellschaft für Krankenhaushygiene. *Bauliche und funktionelle Anforderungen an Eingriffsräume. Leitlinie der Deutschen Gesellschaft für Krankenhaushygiene*. Hygiene & Medizin. 2021. 46: S. 52−56.

35. Halpern, N.A. and Anderson, D.C., *Keeping a 2009 Design Award-Winning Intensive Care Unit Current: A 13-Year Case Study.* HERD. 2020. 13: S. 190−209.

36. Ausschuss für Arbeitsstätten beim Bundesministerium für Arbeit und Soziales. *Technische Regeln für Arbeitsstätten Raumabmessungen und Bewegungsflächen ASR A1.2.* 2022. Zuletzt heruntergeladen [14.10.2022]; Geladen von:https://www.arbeitssicherheit.de/schriften/dokument/0%3A5823941%2C1%2C20130905/?query=ASR%20A1.2.

37. Ausschuss für Arbeitsstätten beim Bundesministerium für Arbeit und Soziales. *Technische Regeln für Arbeitsstätten Sanitärräume ASR A4.1.* 2022. Zuletzt heruntergeladen [14.10.2022]; Geladen von: https://www.arbeitssicherheit.de/schriften/dokument/0%3A5823944%2C1%2C20130905/?query=ASR%204.1.
